# An allosteric propofol-binding site in kinesin disrupts kinesin-mediated processive movement on microtubules

**DOI:** 10.1074/jbc.RA118.002182

**Published:** 2018-05-29

**Authors:** Kellie A. Woll, Stephanie Guzik-Lendrum, Brandon M. Bensel, Natarajan V. Bhanu, William P. Dailey, Benjamin A. Garcia, Susan P. Gilbert, Roderic G. Eckenhoff

**Affiliations:** From the ‡ Department of Anesthesiology and Critical Care and; the ¶ Department of Biochemistry and Biophysics, Epigenetics Program, University of Pennsylvania, Perelman School of Medicine, Philadelphia, Pennsylvania 19104,; the § Department of Biological Sciences and the Center for Biotechnology and Interdisciplinary Studies, Rensselaer Polytechnic Institute, Troy, New York 12180, and; the ‖ Department of Chemistry, University of Pennsylvania School of Arts and Sciences, Philadelphia, Pennsylvania 19104

**Keywords:** microtubule, kinesin, anesthesia, single-molecule biophysics, photoaffinity labeling, molecular motor, tubulin, propofol, single molecule motility

## Abstract

Microtubule-based molecular motors mediate transport of intracellular cargo to subdomains in neurons. Previous evidence has suggested that the anesthetic propofol decreases the average run-length potential of the major anterograde transporters kinesin-1 and kinesin-2 without altering their velocity. This effect on kinesin has not been observed with other inhibitors, stimulating considerable interest in the underlying mechanism. Here, we used a photoactive derivative of propofol, *meta*-azipropofol (AziP*m*), to search for potential propofol-binding sites in kinesin. Single-molecule motility assays confirmed that AziP*m* and propofol similarly inhibit kinesin-1 and kinesin-2. We then applied AziP*m* in semiquantitative radiolabeling and MS microsequencing assays to identify propofol-binding sites within microtubule–kinesin complexes. The radiolabeling experiments suggested preferential AziP*m* binding to the ATP-bound microtubule–kinesin complex. The photolabeled residues were contained within the kinesin motor domain rather than at the motor domain–β-tubulin interface. No residues within the P-loop of kinesin were photolabeled, indicating an inhibitory mechanism that does not directly affect ATPase activity and has an effect on run length without changing velocity. Our results also indicated that when the kinesin motor interacts with the microtubule during its processive run, a site forms in kinesin to which propofol can then bind and allosterically disrupt the kinesin–microtubule interaction, resulting in kinesin detachment and run termination. The discovery of the propofol-binding allosteric site in kinesin may improve our understanding of the strict coordination of the motor heads during the processive run. We hypothesize that propofol's potent effect on intracellular transport contributes to various components of its anesthetic action.

## Introduction

Neurons are highly polarized cells with numerous complex cellular subdomains, such as pre- and post-synaptic termini, which have specialized roles in electrochemical signaling. Because the biosynthetic and degradative machinery reside in the cell body, neurons are uniquely dependent on microtubule-based intracellular transport to deliver their vesicles, organelles, proteins, and RNA to synapses located a meter or more away (reviewed in Refs. 1–4). The human kinesin superfamily includes 45 genes, 38 of which are expressed in brain (5). Three subfamilies of kinesins are predominantly responsible for the fast (∼2–5 μm/s) (6, 7) and slow (∼0.02–0.09 μm/s) (8) transport of cargo to synapses. In contrast, cytoplasmic dynein is the major molecular motor responsible for retrograde transport to the cell body (2, 3, 9, 10).

**Figure 1.**
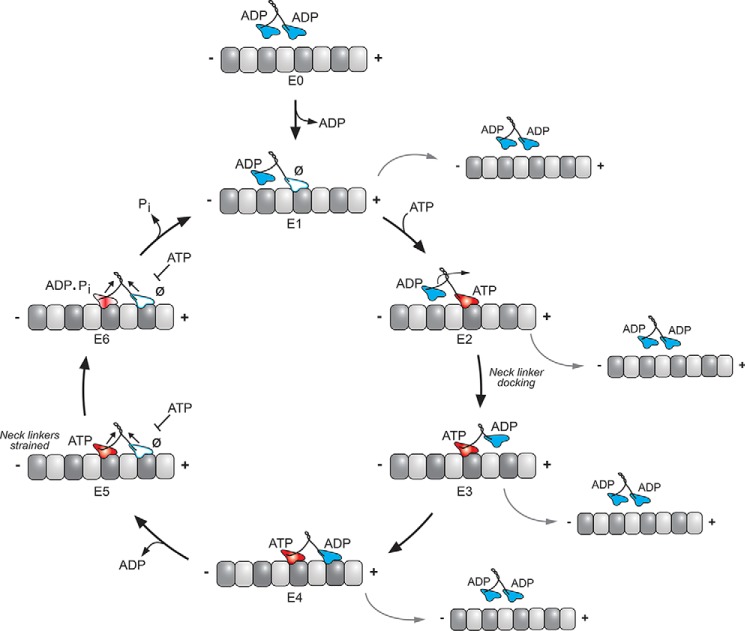
**Generalized schematic of the kinesin stepping cycle with proposed states for propofol-induced premature detachment from the microtubule.** *E0,* dimeric kinesin in solution, detached from the microtubule holds ADP tightly bound in each motor head. *E0–E1,* the processive run starts with microtubule collision followed by ADP release. The leading head is in the no-nucleotide state (*white*; Ø) and the trailing head is detached from the microtubule with ADP tightly bound. *E2–E4*, ATP binding at the leading head initiates a series of structural transitions, namely the ridged movement of the P-loop and Switch I/II motor head subdomains and neck linker docking that promotes the trailing head to move forward to the next microtubule-binding site. *E4–E5*, ADP is released from the leading head resulting in a two-head bound state. Strain develops, the leading head neck linker is detached and pointed backward, which decreases the probability of ATP binding to the leading nucleotide-free head. *E5–E1*, ATP hydrolysis within the trailing head followed P_i_ release weakens the affinity of the trailing head to the microtubule, resulting in its subsequent detachment. The leading head is now able to bind another ATP to continue the processive run. The states most vulnerable to propofol-induced detachment from the microtubule include E1–E4 based on the AziP*m* photolabeling.

The transport kinesins are dimeric with two catalytic motor domains dimerized through a coiled-coil stalk with globular C-terminal domains. The C-terminal domains interact with specific adaptors for cargo binding (reviewed in Refs. 2–4, 11, and 12)). Although conventional kinesin-1 (KIF5 in humans) is homodimeric (13, 14), kinesin-2 KIF3AB and KIF3AC are heterodimeric products of three genes, *KIF3A*, *KIF3B*, and *KIF3C* (15–19). The transport kinesins move along the microtubule in a precise manner in which each ATP turnover is coupled to an 8-nm step, the distance between adjacent αβ-tubulin dimers along the microtubule lattice (20–22). Remarkably, kinesin can complete one hundred steps or more in an asymmetric hand-over-hand manner, and therefore is referred to as “processive” (23–25). The ATPase cycles of each kinesin head must be coordinated and remain out-of-phase with each other to continue a processive run. If both heads reach a microtubule weak binding state at the same time, the processive run ends, and the motor with its cargo detaches from the microtubule (Fig. 1).

**Figure 2.**
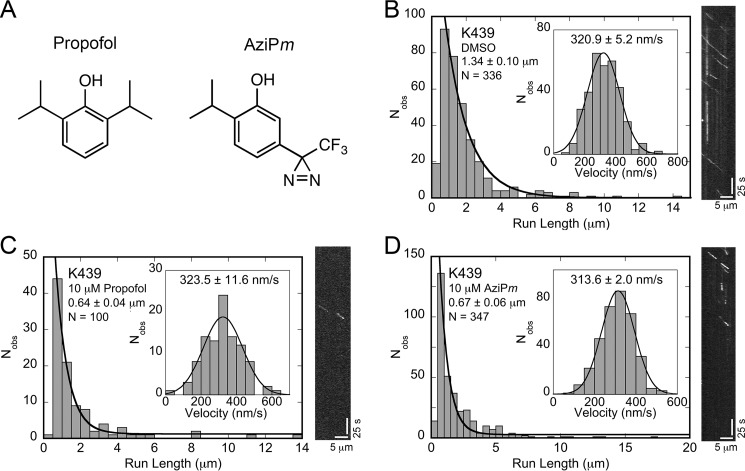
**AziP*m*-like propofol disrupts kinesin-1 processivity without an impact on velocity.** *A,* comparison of the chemical structure of propofol with its photoaffinity derivative, AziP*m. B–D,* single molecule K439 run length and velocity (*inset*) data and representative kymographs of Qdot-labeled K439 motors in (*B*) 5% DMSO control, (*C*) 10 ìM propofol, and (*D*) 10 μm AziP*m*. A single exponential decay fit provides the mean run length ± S.E. for each dataset. Mean run-length differences between the DMSO control and either propofol or AziP*m* were highly significant (*p* ≪ 0.0001), yet the mean run lengths of propofol and AziP*m* conditions showed no statistical significance from each other (*p* > 0.3). A Gaussian fit provides the mean velocity ± S.E. for each dataset, which were not statistically significant between the DMSO control and either propofol or AziP*m* (*p* > 0.1). Kymograph scale bars: 5 μm along the *x* axis, 25 s along the *y* axis.

Previously, we reported that the commonly administered general anesthetic propofol (Fig. 2*A*) at clinically relevant concentrations inhibits the processive movement of kinesin-1 KIF5B and kinesin-2 KIF3AB and KIF3AC (26). The single molecule results showed that the average distance these kinesins could step decreased 40–60% with EC_50_ values <100 nm propofol with no effect on the velocity of movement. These results indicate that propofol is not binding at the ATP-binding site or allosteric sites that modulate microtubule-activated ATP turnover. Rather, the results suggest that during a processive step on the microtubule, an allosteric binding site for propofol forms, propofol binds and disrupts the kinesin–microtubule interaction, and therefore results in kinesin detachment to end the processive run (26).

These results led us to hypothesize that propofol's effect on the neuronal transport kinesins may contribute to the multiplex nature of propofol induction and emergence (27) and/or adverse effects. Although an isolated processivity effect on some kinesins may not translate to large cellular or organism effects, the impact might be larger with prolonged exposures, such as in total intravenous administration (TIVA), ICU sedation, or in particularly sensitive brain regions and/or cell types.

To define the underlying molecular mechanism by which propofol ends a processive run, we pursued a study to identify propofol-binding site(s) on the microtubule–kinesin complex and determine their nucleotide-state dependence. The identification strategy used a photoactive analogue of propofol, *meta*-azipropofol (AziP*m*)^2^ (Fig. 2*A*), coupled with high-resolution MS (28). The residues photolabeled by AziP*m* were located in the motor domains of kinesin-1 and kinesin-2 KIF3B and KIF3C. Interestingly no residues were photolabeled in the KIF3A polypeptide of heterodimeric KIF3AB or KIF3AC. Moreover, the shared allosteric site identified in each was distinct from the ATP-binding site at the conserved Switch I/II subdomain that is highly dynamic over the course of the kinesin stepping cycle (14, 29–33). These results identify a new druggable site in the kinesin family and provide insight into the potential effects of anesthetics on intracellular transport.

## Results

### Alkylphenol-based anesthetics selectively impair kinesin-1 and kinesin-2 run-length potential

We first sought to confirm that the photoaffinity derivative for propofol, AziP*m*, displayed activity analogous to the parent drug propofol. We employed a quantum-dot (Qdot) single molecule assay in conjuction with total internal reflection fluorescence (TIRF) microscopy to evaluate AziP*m* effects on processive kinesin motility (26). The single-molecule motility assay allows quantitative assessment of a motor's run length and velocity of movement by tracking single Qdot-bound kinesin dimers as they step along stationary microtubules (Fig. S1). We examined the effects of AziP*m* on the motility of a bacterially expressed homodimeric conventional kinesin-1 (K439) that encodes the first 439 amino acid residues of human KIF5B. Homodimeric KIF5 motors have been identified as one of the primary motors for anterograde axonal transport of various cargos including vesicles and organelles such as mitochondria (2, 34). Both propofol and AziP*m* significantly decreased the run-length potential of K439 by ∼50% without altering motor velocity (*p* > 0.1) (Fig. 2, *B–D*). This unique disruption in processivity aligned with our previous study where propofol decreased the distance traveled by the well-studied kinesin-1 motor K560 (26), contains the first 560 amino acid residues of KIF5B.

We then extended our study to determine whether AziP*m*, like propofol, also altered the processive movement of kinesin-2 motors. Heterodimeric KIF3 motors have been shown to be important for neurite building and the anterograde component for late endosomes/lysosome bidirectional axonal transport (35–37). Similar to propofol, AziP*m* decreased the run lengths of heterodimeric kinesin-2 KIF3AB and KIF3AC (Fig. S1). Previously we determined that propofol exhibited a level of selectivity for affecting kinesin-2 motors. Namely, propofol decreased the run length of engineered homomeric KIF3BB and KIF3CC but showed no effect on a similarly designed KIF3AA (26). AziP*m* displayed a similar selective inhibition of KIF3BB and KIF3CC motors at 10 μm, decreasing their run lengths by ∼50 and ∼35%, respectively, whereas no significant change in the velocity of movement or the run length was observed for KIF3AA (Fig. S1). Together, these findings indicate that both propofol and the photoaffinity derivative AziP*m* have the same selective disruption of kinesin processive movement likely reflecting a shared molecular mechanism.

### Alkylphenol-based anesthetics bind specifically to the kinesin-1 and kinesin-2 motor domains in the presence of AMPPNP

Processive movement requires that one of the two motor heads of the kinesin dimer be strongly bound with the microtubule at any given time, otherwise the motor would fully detach from the microtubule when under load (38). The strongly bound states within the kinesin stepping cycle occur when the motor head, in complex with tubulin, is bound with ATP or without nucleotide altogether (Fig. 1). We hypothesized that propofol and AziP*m* would disrupt processive movement by interfering with one or both of the strongly bound states resulting in premature detachment from the microtubule. The nonhydrolysable ATP analogue, AMPPNP “locks” processive kinesins in a state similar to that thought for ATP-bound kinesin (39, 40). Therefore to examine potential mechanisms, we performed our studies with microtubule–kinesin complexes with or without AMPPNP to generate a system dominated by either the strongly bound nucleotide-free state or the ATP-bound state within the kinesin stepping cycle. To allow for semi-quantitative analysis of photolabeling of kinesin or tubulin and kinesin–tubulin complexes with or without AMPPNP we performed radiolabeling studies with tritiated AziP*m* ([^3^H]AziP*m*) (41, 42). We irradiated samples containing 10 μm tubulin (microtubule) and 5 μm kinesin with or without 1 mm AMPPNP in the presence of 10 μm [^3^H]AziP*m* with or without 400 μm unlabeled propofol (to determine specificity). Following UV exposure, the proteins were precipitated with acetone, separated by SDS-PAGE, and visualized by Coomassie stain. Protein bands were excised and counted for radiolabel incorporation by scintillation counting (41) (Fig. S2). These values were normalized by the optical density of the Coomassie-stained protein band to prevent unwanted variation.

An increase in [^3^H]AziP*m* radiolabeling was observed for K439, KIF3AB, and KIF3AC in the presence of microtubules indicating the formation of binding sites associated with the microtubule–kinesin complexes rather than kinesin or microtubules alone. Radiolabeling was further increased with the introduction of 1 mm AMPPNP (Table 1). There was a 2–3-fold increase of the kinesin radiolabel photoincorporation in the microtubule–kinesin complexes in the presence of 1 mmAMPPNP compared with microtubule–kinesin complexes without the added nucleotide. To determine the specificity of [^3^H]AziP*m* binding, an excess of 400 μm propofol was included for each assay condition. Addition of 400 μm unlabeled propofol inhibited [^3^H]AziP*m* incorporation by 50–70% indicating that the site(s) present within the microtubule–kinesin–AMPPNP complexes show saturable binding and are shared between the [^3^H]AziP*m* photolabel and parent drug propofol. Considerable concentrations of parent drug are generally used to determine the specificity of binding sites due to the nonequilibrium nature of photoaffinity labeling (42, 43). Indeed over two times greater concentration of propofol was required to reduce labeling in the established protein target, the GABA type A (GABA_A_) receptor, to a similar degree as that used here for kinesin (44).

**Table 1:** **[^3^H]AziPm photoaffinity radiolabeling of kinesin and microtubule–kinesin (MT–kinesin) complexes with and without AMPPNP and unlabeled propofol** The MT–kinesin complex (10 μm MTs + 5 μm kinesin dimer) was preformed with or without (Ø) 1 mm AMPPNP and with (+ propofol) or without (− propofol) 400 μm unlabeled propofol. Values are shown as mean ± S.E. of three experimental replicates.

	Kinesin	MT–kinesin (Ø)		MT–kinesin + AMPPNP	
		− Propofol	+ Propofol	− Propofol	+ Propofol
	*dpm/(OD* × *mm^2^)*	*dpm/(OD* × *mm^2^)*		*dpm/(OD* × *mm^2^)*	
K439	185.8 ± 39.25	304.3 ± 64.96	271.8 ± 50.78	702.8 ± 100.6	345.1 ± 61.33
KIF3AB	146.7 ± 25.18	165.9 ± 39.55	179.7 ± 32.0	458.2 ± 104.9	195.1 ± 44.82
KIF3AC	154.5 ± 37.39	197.7 ± 35.49	232 ± 43.76	559.6 ± 97.62	221.6 ± 38.94

[^3^H]AziP*m* radiolabeling of microtubules across all conditions was less when compared with kinesins (Table 2). Only when microtubules were combined with KIF3AB and 1 mm AMPPNP was there a similar increase in tubulin photoincorporation (∼1.9 fold; Table 2) as compared with radiolabel incorporation into kinesin. Furthermore, the addition of excess unlabeled propofol produced a trend toward inhibition of tubulin radiolabeling in the microtubule–KIF3AB complexes, suggesting a saturable site (Table 2). Interestingly minor increases in radiolabeling of tubulin were observed with addition of 400 μm propofol in KIF3AC and K439-containing systems indicative of either lower affinity and/or nonspecific binding. As a whole, the increases in [^3^H]AziP*m* photolabeling of kinesin and the propofol inhibition of the photoincorporation suggest a specific and/or high affinity kinesin-binding site for propofol. This site appears to be dependent on the formation of the AMPPNP-promoted microtubule–kinesin complex where kinesin is strongly bound to the microtubule.

**Table 2:** **[^3^H]AziPm photoaffinity radiolabeling of microtubules and microtubule–kinesin (MT–kinesin) complexes with and without AMPPNP and unlabeled propofol** The MT–kinesin complex (10 μm MTs + 5 μm kinesin dimer) was preformed with or without (Ø) 1 mm AMPPNP and with (+ propofol) or without (− propofol) 400 μm unlabeled propofol. Values are shown as mean ± S.E. of three experimental replicates.

	Microtubule	MT–kinesin (Ø)		MT–kinesin + AMPPNP	
		− Propofol	+ Propofol	− Propofol	+ Propofol
	*dpm/(OD* × *mm^2^)*	*dpm/(OD* × *mm^2^)*		*dpm/(OD* × *mm^2^)*	
Tubulin (K439)	106.4 ± 37.25	129.8 ± 49.51	152.6 ± 46.4	174.1 ± 73.09	216.2 ± 59.21
Tubulin (KIF3AB)	88.97 ± 24.0	154.6 ± 28.2	178.9 ± 12.9	297.4 ± 48.07	185.5 ± 50.5
Tubulin (KIF3AC)	65.1 ± 17.19	103.7 ± 29.95	151.1 ± 50.2	148.6 ± 22.66	137 ± 28.5

### Alkylphenol-based anesthetic-binding sites within the K439, KIF3B, and KIF3C catalytic motor head domains

**Figure 3.**
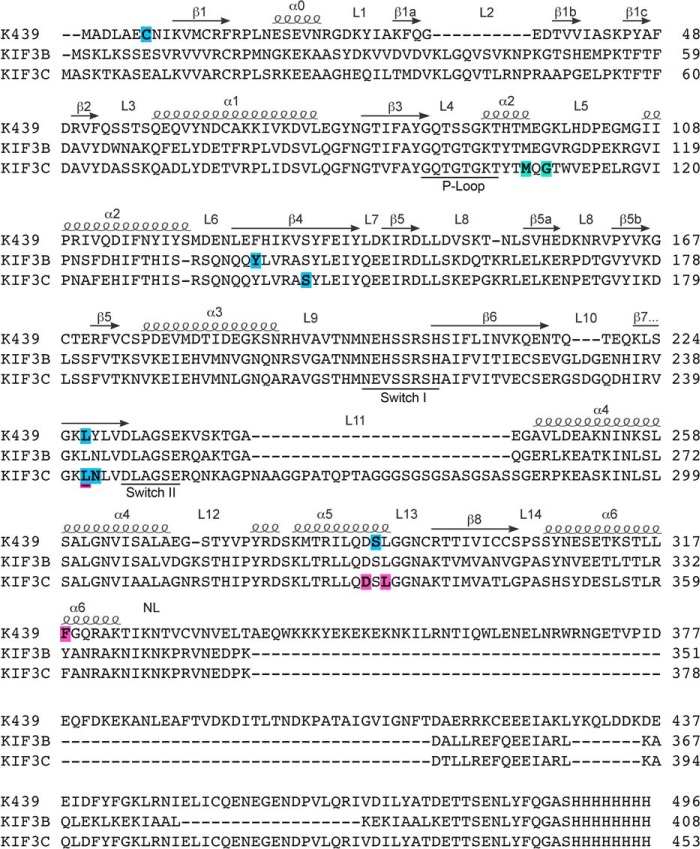
**Photolabeled residues in kinesin-1 and kinesin-2 motor domains.** Shown is alignment of kinesin motor domain sequences from kinesin-1 (K439) and kinesin-2 (KIF3B and KIF3C), with structural elements from kinesin-1 (KIF5B, PDB code 4HNA) provided above the alignment (30). Residues highlighted in *magenta* were photolabeled by AziPm in the no-nucleotide state for K439 (Phe^318^) and KIF3C (Leu^242^, Asp^330^, Leu^332^). Residues in the ATP-like AMPPNP-bound state within the common allosteric binding site are highlighted in *cyan* for K439 (C7, Leu^227^, Ser^289^), KIF3B (Tyr^138^), and KIF3C (Ser^144^, Leu^242^, Asn^243^). The KIF3C residue Leu^242^ was photolabeled by AziP*m* in the absence of nucleotide and in the presence of 1 mm AMPPNP, the ATP-like state (*cyan* with *magenta underline*). Additionally, residues photolabeled at a site unique to KIF3C in the presence of AMPPNP (Met^107^, Gly^109^) are highlighted in *teal*.

We next identified the AziP*m* photolabeled residues within kinesin and/or tubulin of the microtubule–kinesin complexes with or without AMPPNP by mass spectrometry (MS) microsequencing. All kinesins, with or without AMPPNP, showed ample sequence coverage (80–96%) with K439 displaying 95 and 96% coverage in conditions with and without AMPPNP, respectively (Fig. S3–S6). Major isoforms of α (TBA4A)- and β (TBB4B)-tubulin showed >75% coverage across all experiments (Figs. S7 and S8). An increased number of AziP*m* adducts were identified in K439 in the microtubule–K439–AMPPNP complexes compared with the complexes with no added nucleotide (Fig. 3; Fig. S9). This trend was also observed for the KIF3B and KIF3C motors when the microtubule KIF3AC and KIF3AB complexes were photolabeled with or without AMPPNP (Fig. 3; Fig. S10–S12). All residues photolabeled by AziP*m* in KIF439, KIF3B, and KIF3C were located in the motor domain (Fig. 3). Interestingly, no AziP*m* adducts were detected within KIF3A peptides across all experiments. These observations suggest that the lack of inhibition by propofol and AziP*m* for homodimeric KIF3AA in single molecule motility studies was a result of KIF3A not binding propofol or AziP*m*. Moreover, the results indicate that KIFA in KIF3AB and KIF3AC was also unable to bind propofol or AziP*m*.

### AMPPNP-dependent formation of propofol-binding sites common between kinesin motor domains

To explore the results of our photolabeling experiments for each microtubule–kinesin complex, we aligned the kinesin sequences with the X-ray crystal structures of the kinesin motor domain without nucleotide (PDB ID 4LNU) (29) or with adenosine diphosphate-aluminum fluoride (ADP-AlF_4_^−^) (PDB ID 4HNA) (30) in complex with tubulin (Fig. S13). To further clarify propofol and AziP*m*-binding cavities, we substituted residues within kinesin motor head X-ray crystal structures to match the K439 sequence and analyzed both structures with Computed Atlas of Surface Topography of proteins (CASTp) web software (45). CASTp predicts binding cavities within proteins and provides the measurements as well as the contributing amino acid residues for the detected binding pockets. Pockets of sufficient volume to accommodate at least one propofol or AziP*m* molecule (>200 Å^3^) (Fig. S14) were investigated with docking experiments using AutoDock Vina (46) to further refine potential binding sites. The docking regions were set to include all amino acid residues that were predicted to contribute at least one atom of the side chain or backbone to the binding pocket lining. Additionally, all residue side chains were made flexible within the docking experiment to provide an estimate of ligand binding energies. Within each structure, atoms from the majority of photolabeled residues detected by MS in the microtubule–kinesin complexes with or without AMPPNP were found to line computationally defined binding pockets with appropriate volumes.

**Figure 4.**
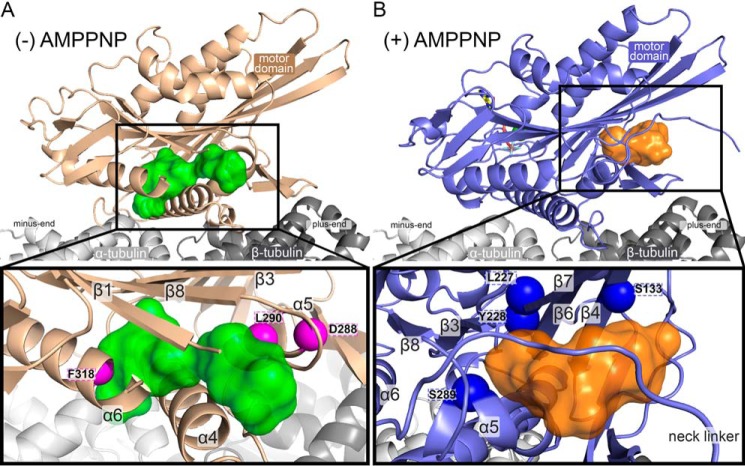
**Location of predicted propofol and AziP*m*-binding pockets and associated photolabeled residues in microtubule–kinesin complexes without and with AMPPNP.** *A,* side and focused view of the X-ray crystal structure of kinesin motor head in complex with tubulin in the absence of nucleotide (PDB ID 4LNU) (29) respresenting photolabeled microtubule–kinesin complexes without (−) AMPPNP. The *green* Connolly surface representations highlight the highest scored poses for five propofol and five AziP*m* as predicted by AutoDock Vina (46) within the CASTp (45) predicted binding cavity (see appendix Fig. S11). *Magenta spheres* indicate the α-carbon atoms of Phe^318^ photolabeled in K439 and Asp^288^ and Leu^290^ that correspond with the photolabeled residues Asp^330^ and Leu^332^ in KIF3C, respectively. *B,* side and focused view of the X-ray crystal structure of ADP-AlF_4_^−^-bound kinesin motor head in complex with tubulin (PDB ID 4HNA) (30) representing photolabeled microtubule–kinesin complexes with (+) AMPPNP. The *orange* Connolly surface representations highlight five propofol and five AziP*m* in the highest scored poses predicted by AutoDock Vina (46) within the CASTp (45) predicited binding cavity (see Fig. S14). *Blue spheres* indicate the α-carbon atoms of the photolabeled residue in K439 (Ser^289^), photolabeled residues in KIF3C (Ser^144^ and Asn^243^) corresponding with Ser^133^ and Tyr^226^ in K439, respectively, and the photolabeled residue in both K439 (Leu^227^) and KIF3C (corresponding with Leu^242^). ADP-AlF_4_^−^ is shown in stick representation and a Mg^2+^ ion is shown as a *light green sphere*. All residues within predicted binding sites were made flexible in AutoDock Vina, whereas the backbone structure remained rigid during docking experiments.

Docking within the kinesin motor domain of the tubulin–kinesin complex without nucleotide indicated that an interior pocket between the α4 and α6 helices and β-strands β3, β7, and β8 would accommodate propofol and AziP*m* with poses represented within the site (Fig. 4*A*). A continuous solvent accessible pocket was also indicated between the α4 and α5 helices and β-strands β1 and β3 for both ligands (Fig. 4*A*). Photolabeled residues identified within microtubule–kinesin complexes without nucleotide are in close proximity to the highest scored poses. Specifically photolabeled residues located at α5 and α6 align well with both the CASTp-predicted binding site and distances from highest scored poses generated by AutoDock Vina (Fig. 4*A*). Propofol is expected to be highly dynamic in its binding site, and thus more than one pose is likely to reflect reality, and is the basis for multiple photolabeled residues.

Significant conformational changes within the motor head are observed when comparing the no nucleotide and ADP-AlF_4_^−^-bound kinesin X-ray crystal structures (29). These structural changes are generally described as rigid movements involving three distinct subdomains: P-loop and Switch I/II subdomains, and the microtubule-binding interface (29). Namely, when the microtubule–kinesin complex binds ATP, the nucleotide cleft closes resulting in concerted rotations of the P-loop and Switch I/II subdomains (29, 31). The movement of the subdomains results in the obstruction of the interior pocket that was predicted by CASTp in the no nucleotide-bound kinesin motor. Instead, within the ADP-AlF_4_^−^-bound microtubule–kinesin complex, CASTp predicted a large cavity located mainly between the α5 helix, β-strands β3, β4, β6, and β7 and the neck linker (Fig. S14). Propofol and AziP*m* displayed similar poses in the cavity, with the highest scored poses by AutoDock Vina within the α5 helix and β-strands β4, β6, and β7 (Fig. 4*B*). Again photolabeled residues by AziP*m* were consistently near poses generated by docking, specifically photolabeled residues located within β-strands β4 and β7 (Fig. 4*B*).

Together photoaffinity labeling experiments provide evidence that alkylphenol-based anesthetics bind within microtubule–kinesin complexes both in the presence and absence of AMPPNP. Indeed [^3^H]AziPm radiolabeling, which provides a quantitative assessment of photolabeling, aligns with these findings. Although not significant, photoincorporation may be increased within microtubule–kinesin complexes without nucleotide compared with samples only containing dimeric kinesin. However, given that [^3^H]AziPm photoincorporation was comparatively lower and propofol did not inhibit the radiolabeling within complexes without nucleotide suggests that the pocket identified within AMPPNP-bound microtubule–kinesin complexes better represents a higher affinity and/or propofol-specific site. An additional compelling feature was the photolabeling of similar putative binding pockets identified within the AMPPNP-bound complex relative to no nucleotide-bound microtubule–kinesin complex, namely those photolabeled residues within α5 helix and β-strands β1 and β7 (Fig. S13). These results suggest that alkylphenol-based anesthetics may also bind to an intermediate state between the two static crystal structures represented. Additional studies and increased understanding of these intermediate conformations will be needed to test this potential mechanism.

### Additional kinesin-specific alkylphenol-based anesthetic binding sites

**Figure 5.**
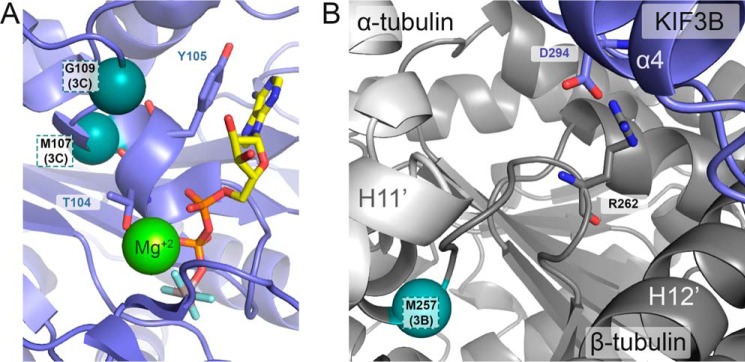
**AziPm photolabeled residue identified in secondary sites for KIF3C and KIF3AB β-tubulin within the AMPPNP-bound microtubule–kinesin complexes.** *A* and *B,* expanded views of the X-ray crystal structure of ADP-AlF_4_^−^-bound kinesin motor head in complex with tubulin (PDB ID 4HNA) to represent the microtubule–kinesin complexes with AMPPNP. *A,* photolabeled residues (Met^107^ and Gly^109^) within the KIF3C second the photolabeled pocket from the nucleotide-binding site are labeled accordingly. ADP-AlF_4_^−^ is shown in stick representation and a magnesium ion is shown as a *light green sphere. B,* photoaffinity labeled residue (Met^257^) in β-tubulin within KIF3AB microtubule–kinesin complexes with AMPPNP and the residue's relationship to TUBB3 Arg^262^. The α-carbon of photolabeled residues are represented as *teal spheres* and are labeled accordingly.

An additional site was photolabeled by AziP*m* in KIF3C (Met^107^ and Gly^109^) located at an interface of the P-loop and Switch I/II domains that were near, but not contributing to, the nucleotide-binding site (Fig. 5). Furthermore, despite the comparative abundance and proximity to the kinesin motor, only one residue within β-tubulin was photolabeled by AziP*m* and only in the KIF3AB ATP-like system (Met^257^; Fig. 5). This residue is close to the human TUBB3 R262H and R262A mutation sites that impair the motility and ATPase activity of the kinesin motor (47–50). It is not clear how these secondary sites contribute to AziP*m* and propofol's mechanism(s), however, they may hint toward additional kinesin-specific effects. Ultimately, the conserved site shared by K439, KIF3B, and KIF3C at the junction of Switch I/II subdomain and microtubule-binding interface is likely to be the main contributor to the common effects of alkylphenol-based anesthetics to decrease kinesin run length, whereas preserving velocity.

## Discussion

This study indicates that propofol binds at a site allosteric to the catalytic site within the kinesin motor head, which forms in the ATP-bound state, leading to premature detachment of anterograde processive kinesins and their cargos from the microtubule (Fig. 1). To our knowledge, this common site identified in K439, KIF3B, and KIF3C is the first evidence of a druggable inhibitory site within the interface of the Switch I/II subdomain and the microtubule–kinesin interface. Given that the mechanism of kinesin processivity appears to rely on the collective movement of these subdomains, the binding of inhibitors at their interfaces would be a rational mechanism to alter the kinesin motor head internal reorganization and impair motor head coordination for the kinesin stepping cycle. Indeed, interfacial binding between motor head subdomains has been previously observed for other kinesin allosteric inhibitors (51–53). However, these sites are located at alternative sites within kinesin compared with the common binding site identified for propofol and these inhibitors affect both the velocity and ATPase activity. We hypothesize that propofol binding at the Switch I/II subdomain and microtubule interface would promote the premature formation of a weak binding state that disrupts motor head coordination resulting in motor detachment. Such a mechanism, largely distinct from ATP hydrolysis, would be predicted to result in the decreased kinesin run length, whereas maintaining normal velocity. Furthermore, if propofol binding occurred when only one motor head was in complex with the microtubule during the kinesin mechanochemical cycle, then there would be an increased likelihood of premature detachment (*E2* and *E3*, Fig. 1). More investigations into the structure and/or kinetics of propofol binding within specific kinesins and their intermediate states will help to resolve these subtle mechanistic distinctions.

The nanomolar potency to inhibit kinesin processivity is in contrast to other functionally influenced protein targets identified for propofol. Indeed affinities for many binding targets and the plasma concentrations often associated with propofol are within the low micromolar range. Many general anesthetics are considered hydrophobic drugs, with propofol water/octanol partition coefficient of ∼3.79. As a consequence it is likely that propofol concentrates in more hydrophobic biological microdomains such as lipid bilayers. We anticipate that the conditions of the Qdot single molecule assay would be reflective of the cytosol and therefore the significant inhibition of kinesin processivity at nanomolar concentrations would, within a biological context, be clinically relevant. Further studies to determine the impact on cellular, or more likely, the neuronal circuit are required to evaluate the contribution of this effect on propofol's mechanism of action.

Previously we showed that the general anesthetic propofol significantly reduced the run-length potential of processive kinesins without altering their velocity of movement (26). Additionally, this study demonstrated that the decrease was specific to the propofol 1-hydroxyl, because a fluorine-substituted derivative lacking anesthetic activity also had no effect on kinesin processivity. The current study confirms that the photolabel analogue AziP*m* has a similar unique functional activity to propofol, and connects this activity to a specific allosteric binding site within kinesin. The hypnotic effects of general anesthetics like propofol are typically attributed to ligand-gated ion channels, such as the GABA_A_ receptor (54). However, studies on mice lacking various subunits of the GABA_A_ receptor still exhibit a hypnotic response to anesthetic exposure. Furthermore, the data that have been reported for various other molecular targets are insufficient to establish these ion channels as both necessary or sufficient to produce all the various components that constitute the state of anesthesia, as well as the many adverse effects (55).

Propofol's potent ability to cause a 40–60% reduction in kinesin run length (26) provides an additional molecular mechanism for altering neuronal function: a disruption of intracellular transport. Indeed various mechanisms that require kinesin, including neurite growth and specification as well as vesicle exocytosis, are influenced by anesthetics (56, 57). Nevertheless, an important physiological role for this propofol–kinesin interaction remains to be demonstrated. In summary, this work provides important structural clues of a novel molecular mechanism for disrupting kinesin processivity and broadens the prospective targets involved in propofol pharmacology.

## Experimental procedures

### Kinesin-1 K439 cloning and expression

The expression construct for human kinesin-1 K439 was a custom gene synthesis from GenScript (Piscataway, NJ) as an insert in the pET24d expression vector between NcoI and BamHI restriction sites. This construct encoded the first 439 residues of *Homo sapiens* KIF5B, a C terminally fused EB1 sequence that matched the coiled-coil registry of the helix to create stable dimers (bold), a TEV protease site (italicized) with linker residues (plain font), and a His_8_ tag (underlined): K439, *Hs* KIF5B (Met^1^–Ile^439^)-**DFYFGKLRNIELICQENEGENDPVLQRIVDILYATDE***TTSE; NLYFQ*GASHHHHHHHH (predicted *M*_r_ = 56,538).

K439 was bacterially expressed based on the protocol for kinesin-1 K560 as previously reported (26). Briefly, *Escherichia coli* BL21-CodonPlus (DE3)-RIL cells (Stratagene, La Jolla, CA) were transformed with the K439 plasmid followed by selection on lysogeny broth (LB) plates containing 50 μg/ml of kanamycin and 10 μg/ml of chloramphenicol. Positive colonies were selected and grown in LB liquid culture containing the same selective antibiotics at 37 °C until they reached *A*_600_ of 0.4–0.5. Cultures were then chilled on an ice bath to 16 °C before expression was induced by the dropwise addition of 0.1 mm isopropyl β-d-1-thiogalactopyranoside (IPTG), which continued for ∼16–18 h at 16 °C shaking at 185 rpm.

### Kinesin-2 KIF3 cloning and expression

Preparation of mouse KIF3AB constructs was described in detail previously (58). Briefly, each expression plasmid encoded the native N-terminal motor domain sequence through the native helix α7 followed by a C terminally fused synthetic heterodimerization helix (SHD; bold) motif containing either an acidic (AHD) or basic (BHD) sequence that generated pairwise stabilized heterodimeric motors. The SHD was followed by a TEV protease site (italicized) with linker residues (plain font) and affinity purification tags (underlined). The use of a StrepII tag on KIF3A-AHD combined with the use of a His_8_ tag on KIF3B-BHD provided the ability to purify stable heterodimers with sequential affinity purification steps: KIF3A(Met^1^–Glu^376^)-**LEKEIAALEKEIAALEK**TTS*ENLYFQ*GASNWSHPQFEK (predicted *M*_r_ = 46,341); KIF3B(Met^1^–Lys^371^) **LKEKIAALKEKIAALKE**TTS*ENLYFQ*GASHHHHHHHH (predicted*M*_r_ = 45,790).

Preparation of mouse KIF3AC was described in detail previously (59), but briefly the design replicated the strategy used for KIF3AB with the exception that the native N-terminal motor through α7 was followed by a C terminally fused sequence of EB1 for dimerization (bold) that matched the coiled-coil registry of the native helix, a TEV protease site (italicized) with linker residues (plain font) and affinity purification tags (underlined). As with KIF3AB, the use of the StrepII tag on KIF3A combined with a His_8_ tag on KIF3C allowed for sequential purification steps to generate pure, stable heterodimeric KIF3AC: KIF3A-EB1, KIF3A(Met^1^–Leu^374^)-**DFYFGKLRNIELICQENEGENDPVLQRIVDILYATDE**TTS*ENLYFQ*GASNWSHPQFEK (predicted *M*_r_ = 48,559); KIF3C, KIF3C(Met^1^–Leu^396^)- **DFYFGKLRNIELICQENEGENDPVLQRIVDILYATDE**TTS*ENLYFQ*GASHHHHHHHH (predicted *M*_r_ = 49,759).

This same KIF3C construct was used for generating homodimeric KIF3CC for the experiments reported herein. The constructs used to express KIF3AA and KIF3BB differ from those detailed above for heterodimeric motors. Rather than use SHD for heterodimerization, KIF3A and KIF3B homodimeric clones utilized the EB1 dimerization strategy (bold) described above for KIF3C, a TEV protease site (italicized) with linker residues (plain font), and a His_8_ tag for purification (underlined): KIF3A(Met^1^–Leu^374^)-**DFYFGKLRNIELICQENEGENDPVLQRIVDILYATDE**TTS*ENLYFQ*GASHHHHHHHH (predicted *M*_r_ = 48,502); KIF3B(Met^1^–Leu^369^)-**DFYFGKLRNIELICQENEGENDPVLQRIVDILYATDE**TTS*ENLYFQ*GASHHHHHHHH (predicted *M*_r_ = 48,011).

All KIF3 motors were bacterially expressed as previously reported (26, 58) in *E. coli* BL21-CodonPlus (DE3)-RIL cells (Stratagene). Heterodimeric KIF3AB and KIF3AC resulted from bacteria that were co-transformed by electroporation with the two appropriate motor expression plasmids, followed by selection on lysogeny broth (LB) plates containing 100 μg/ml of ampicillin, 50 μg/ml of kanamycin, and 10 μg/ml of chloramphenicol. Homodimeric KIF3AA, KIF3BB, and KIF3CC each resulted from bacterial transformation with a single homodimer-designed plasmid followed by selection on LB plates containing 100 μg/ml of ampicillin and 10 μg/ml of chloramphenicol. Positive colonies were selected and grown in LB liquid culture containing the same selective antibiotics at 37 °C until they reached *A*_600_ of 0.4–0.5. Cultures were then chilled on an ice bath to 16 °C before expression was induced by the dropwise addition of 0.1 mm IPTG, which continued for ∼16–18 h at 16 °C shaking at 185 rpm.

### Purification of kinesin dimers

For all kinesins reported herein, cultures were subjected to centrifugation to collect cell pellets, which were then resuspended by spinning at 4 °C with the addition of lysis buffer to 10 ml/g of cells. Lysis buffer contained 10 mm sodium phosphate buffer, pH 7.2, 300 mm NaCl, 2 mm MgCl_2_, 0.1 mm EGTA, 0.02 mm ATP, 1 mm DTT, 10 mm phenylmethylsulfonyl fluoride, and 30 mm imidazole. Cells were lysed by the addition of 0.2 mg/ml of lysozyme to the resuspended cells with continued stirring for 50 min at 4 °C, followed by three replicate cycles of freezing in liquid N_2_ and thawing in a 37 °C water bath. Lysate was then clarified by ultracentrifugation and applied to a HisTrap FF Ni^2+^-NTA column (GE Healthcare) that had been pre-equilibrated with Ni^2+^-NTA binding buffer (20 mm sodium phosphate buffer, pH 7.2, 300 mm NaCl, 2 mm MgCl_2_, 0.1 mm EGTA, 1 mm DTT, 0.02 mm ATP, and 30 mm imidazole).

For K439, the loaded column was then washed with K439 Wash Buffer (20 mm sodium phosphate buffer, pH 7.2, 300 mm NaCl, 2 mm MgCl_2_, 0.1 mm EGTA, 1 mm DTT, 0.02 mm ATP, and 50 mm imidazole) until baseline absorbance was established. His-tagged K439 was then eluted with a linear gradient (Ni^2+^-NTA wash buffer: 50–400 mm imidazole, pH 7.2). Positive elution fractions were pooled, concentrated, and initially dialyzed overnight at 4 °C against K439 Dialysis Buffer (20 mm HEPES, pH 7.2, with KOH, 0.1 mm EDTA, 0.1 mm EGTA, 5 mm magnesium acetate, 50 mm potassium acetate, 1 mm DTT plus 300 mm NaCl). Three additional dialysis steps were conducted the following day for 60 min, each at 4 °C to gradually decrease the concentration of NaCl (K439 Dialysis Buffer: 200 mm, 150 and 100 mm NaCl). The final dialysis buffer for K439 at 100 mm NaCl also included 5% sucrose.

The purification of kinesin-2 motors (KIF3AC, KIF3AB, KIF3AA, KIF3BB, and KIF3CC) has been reported previously (26, 58), but briefly the loaded HisTrap Ni^2+^-NTA column was then washed with excess Ni^2+^-NTA binding buffer, and motors were eluted with a linear gradient (Ni^2+^-NTA binding buffer: 30–300 mm imidazole, pH 7.2). For homodimeric motors, positive elution fractions were pooled, concentrated, and dialyzed at 4 °C overnight in 20 mm HEPES, pH 7.2, with KOH, 0.1 mm EDTA, 0.1 mm EGTA, 5 mm magnesium acetate, 50 mm potassium acetate, 1 mm DTT plus 100 mm NaCl, and 5% sucrose. For heterodimeric motors, positive HisTrap Ni^2+^-NTA fractions were pooled and transferred to a StrepIITactin^TM^ column (StrepTrapII HP, GE Healthcare) that had been pre-equilibrated with StrepII column buffer (20 mm sodium phosphate buffer, pH 7.2, 300 mm NaCl, 2 mm MgCl_2_, 0.1 mm EGTA, 1 mm DTT, 0.2 mm ATP). The loaded StrepII column was washed with excess StrepII column buffer to return to baseline absorbance, followed by elution in StrepII column buffer plus 2.5 mm desthiobiotin. Fractions from the StrepII column were analyzed by SDS-PAGE to identify only fractions containing a 1:1 ratio of each KIF3 polypeptide. These fractions were then pooled, concentrated, and dialyzed at 4 °C overnight in 20 mm HEPES, pH 7.2, with KOH, 0.1 mm EDTA, 5 mm magnesium acetate, 50 mm potassium acetate, 1 mm DTT, 5% sucrose, 100 mm NaCl. The purity of all motors was confirmed by both analytical gel filtration on an HPLC gel filtration column (Superose^TM^ 10/300, GE Healthcare Life Sciences) and SDS-PAGE. Note that the purification tags were not cleaved for the experiments reported herein. Typical yields for purified motors from bacterial expression were 1.7–3 mg/g of *E. coli*. Predicted molecular weights based on amino acid residue sequence are as follows: K439 at 113,076; KIF3AC at 98,317; KIF3AB at 92,131; KIF3AA at 97,000; KIF3BB at 96,022; and KIF3CC at 99,518. Prior to each experiment, kinesin aliquots were thawed, clarified for 10 min at 4 °C (Beckman Coulter TLX Optima Ultracentrifuge, TLA-100 rotor, 313,000 × *g*), and the protein concentration was determined using the Bio-Rad protein assay with IgG as a protein standard.

### Single molecule motility assays

Kinesin motors were visualized by attachment of streptavidin-coated quantum dots (Qdot 525-Streptavidin conjugate, Life Technologies) to the His-tagged C terminus. Qdots were preincubated with biotinylated-Penta-His antibody (Qiagen) at a 1:1 ratio (200 nm each) for 60 min at room temperature in PME80 buffer (80 mm PIPES, pH 6.9, with KOH, 5 mm MgCl_2_, and 1 mm EGTA). To this mixture, His-tagged kinesin dimers were added to a final concentration of 20 nm dimer and incubated for an additional 60 min at 4 °C. This process generated a working stock of Qdot-labeled motors at a ratio of 1:10 kinesin dimer:Qdots (20 nm dimer, 200 nm Qdot–antibody complex). According to a Poisson distribution, 9% of the Qdots in this working stock are estimated to have one motor bound and 0.5% with ≥2 motors bound. Previous KIF3 experiments confirmed that a 1:10 ratio of kinesin motors to Qdots was sufficient for single molecule conditions (59).

Long microtubule tracks (13–30 μm) used for microscopy were polymerized as previously described (59) from lyophilized X-rhodamine tubulin (Cytoskeleton, Inc., Denver, CO). Tubulin was resuspended in PME80 buffer containing 10% glycerol, followed by incubation on ice for 5 min in the presence of 1 mm MgGTP. The tubulin was then centrifuged at 16,000 × *g* for 10 min at 4 °C (Galaxy 16D Micro 1816 centrifuge, VWR, Bridgeport, NJ). The clarified supernatant that contained soluble tubulin was transferred to a new tube, and polymerization was conducted at 37 °C for 20 min. The polymerized microtubules were then stabilized by the addition of paclitaxel (final 33 μm final concentration) and incubated for an additional 10 min. This procedure yields a final microtubule stock at 30 μm tubulin polymer.

Perfusion chambers were formed by mounting a silanized coverslip on a glass slide with strips of double-sided tape to generate a 10-μl flow cell. Chambers were first incubated with 0.4% rat anti-α-tubulin antibody (ABD Serotec, Raleigh, NC) for 5 min followed by surface blocking with 5% Pluronic F-127 (Sigma) for 5 min.

X-rhodamine microtubules (30 μm stock) were diluted 1:300 in PME80 supplemented with paclitaxel to 22 μm to generate a final microtubule concentration at 0.1 μm. Microtubules were then introduced into the perfusion chamber and incubated for 10 min. Unbound microtubules were removed by washing the chamber with excess PME80 supplemented with 10 mm DTT and 20 μm paclitaxel. For kinesin-2 motors, the working stock of Qdot–motor complexes (20 nm dimer/200 nm Qdot–antibody complex) was then added to an Activity Buffer (PME80, 0.5% Pluronic F-127, 30 μm paclitaxel, 125 μg/ml of BSA, 50 μm DTT, 25 mm glucose, 0.2 mg/ml of glucose oxidase, 175 μg/ml of catalase, 0.3 mg/ml of creatine phosphokinase, 2 mm phosphocreatine, 1 mm MgATP, 5% DMSO ± 10 μm AziP*m* or Propofol) to dilute the motors 10 times and give a final concentration of 2 nm Qdot–motor complex in the chamber. Adding Activity Buffer to the perfusion chamber activated motor activity, and chambers were imaged immediately.

For Qdot–K439 complexes, 0.5 mm AMPPNP was required to generate the microtubule–K439–Qdot complex. Therefore, the setup of the perfusion chamber was altered such that immediately following the 10-min microtubule binding step, Qdot–K439 complexes were introduced in PME80 supplemented with 10 mm DTT, 20 μm paclitaxel, and 0.5 mm Mg-AMPPNP and incubated in the chamber for 5 min. This step replaced the wash step to remove unbound microtubules. Qdot–K439 complexes were then activated by introduction of Activity Buffer containing 1 mm MgATP.

### TIRF microscopy and image acquisition

Single molecule motility chambers were imaged by TIRF microscopy at 25 °C using a Zeiss Inverted Axio Observer Z1 MOT fluorescence microscope with the ×100 oil 1.46 N.A. Plan-Apochromat objective (Carl Zeiss Microscopy, Inc., Jena, Germany) and an incubation hood as previously described (59). Digital images were collected with a Hamamatsu electron multiplier EM-CCD digital camera using the AxioVision 4.8.2 software package. The images generated were 512 × 512 pixels with 0.16 μm/pixel in both *x*- and *y*-planes. Qdot–kinesin complexes were tracked by imaging at 488 nm (5% laser power, 100 ms exposure) every ∼0.5 s for 5 min. Reference images of the X-rhodamine microtubule tracks were taken at 564 nm (2% laser power, 300 ms exposure) both before and after acquisition of data in the Qdot channel. Qdot videos were then overlaid with the microtubule image using NIH ImageJ software.

### Data analysis

Single molecule motility was analyzed with ImageJ and the MultipleKymograph plugin (J. Rietdorf and A. Seitz, European Molecular Biology Laboratory, Heidelberg, Germany). Mean velocity ± S.E. was determined from histograms of velocity data with an applied Gaussian function. Mean run lengths were determined from histograms of run length data with an applied single exponential decay fit according to the following equation,

(Eq. 1) $$y=y_{0}+A^{\left(-x/l\right)}$$

where *A* is the maximum amplitude and *l* is the mean run length reported ± S.E. The first bin of run-length histograms was masked from the fit due to the resolution limit of the TIRF microscope (<0.25 μm).

To ensure that the maximum run-length potential was examined, Qdot–kinesin complexes were excluded from analysis if they reached the end of the microtubule before falling off, paused at the microtubule end, or began or ended a run outside the timescale of the experiment. Only long microtubule tracks were used for data analysis, which allowed for collection of both long and short runs on the same tracks, thereby avoiding data bias. Statistical analysis to compare mean values from single molecule datasets was conducted with a Student's *t* test using an α-reliability level of 5%.

### Preparation of microtubules for photoaffinity labeling

Aliquots of purified bovine brain tubulin were diluted with an equal volume of PM buffer (100 mm PIPES, pH 6.9, 5 mm magnesium acetate, 1 mm EGTA) and cold-depolymerized on ice for 30 min in the presence of 1 mm GTP. Tubulin was then clarified by centrifugation at 16,000 × *g* for 15 min at 4 °C and the soluble tubulin supernatant was removed to a fresh tube. Soluble tubulin was then polymerized by incubation at 37 °C, and microtubules were stabilized by the addition of 20 μm Taxol. Microtubules were pelleted at 16,000 × *g* at ambient temperature and resuspended in ATPase buffer (20 mm HEPES, pH 7.2, 5 mm magnesium acetate, 0.1 mm EDTA, 0.1 mm EGTA, 50 mm potassium acetate, 5% sucrose) containing 1 mm DTT and 40 μm Taxol. The concentration of tubulin polymer was determined by Lowry assay with BSA as a protein standard.

### Photoaffinity radiolabeling

Reaction samples contained final concentrations of 10 μm tubulin, 5 μm kinesin dimer, with or without 1 mm Mg-AMPPNP in ATPase buffer containing 1 mm DTT, 40 μm paclitaxel, 10 μm [^3^H]*meta*-azipropofol ([^3^H]AziP*m*), and 6% methanol with or without 400 μm propofol. Reactions were prepared in the following order: 1) ATPase buffer containing DTT and paclitaxel, 2) tubulin dimer, 3) kinesin, 4) AMPPNP or buffer, then 5) [^3^H]AziP*m* in methanol with or without propofol. Immediately after preparation, half of the reaction mixture was placed in a 1-mm path length quartz cuvette and exposed to UV irradiation at 350 nm, RPR-3000 Rayonet lamp through a WG295 295-nm low-pass glass filter (Newport Corporation) for 25 min. The remaining half of the reaction mixture was used as a matched non-UV irradiated control. After UV exposure samples were added to 4 volumes chilled acetone and proteins were precipitated overnight at −20 °C. Protein was pelleted for 20 min at 16,000 × *g* at 4 °C then gently washed twice with 300 μl of chilled acetone. Protein pellets were air-dried before resuspension in 50 μl of 50 mm Tris-HCl, pH 8.0, 1% Triton X-100, and 0.5% SDS. Insoluble debris was pelleted by centrifugation at 16,000 × *g*. The supernatant was removed, and protein content was determined by BCA assay. SDS-PAGE sample buffer at ×5 was added, and samples were heated for 3 min at 90 °C. Total sample protein (1–3 μg) was separated on a 10% SDS-PAGE gel. Gels were stained and fixed in 10% acetic acid, 50% methanol, 0.5% Coomassie Blue G-250 in ddH_2_O and destained with 10% acetic acid + 10% methanol in ddH_2_O. After destaining the gels were washed three times with ddH_2_O. Gels were scanned on a Bio-Rad GS-800 calibrated densitometer with quantification using the accompanying Quantity One software. To correct for the differences in optical density measurements caused by the background staining of an individual gel, the background was subtracted with a box drawn in a blank gel lane adjacent to the sample band and the mean optical density (O.D.) multiplied by the band area was recorded from contoured bands (OD × mm^2^). Representative gel images of protein bands are shown in Fig. S2.

Bands were excised and placed into scintillation vials. One ml of 30% hydrogen peroxide (v/v %) was added, and vials were incubated overnight at 65 °C to dissolve the polyacrylamide. Samples were then cooled to ambient temperature before the addition of 10 ml of EcoLite(+) liquid scintillation mixture. Samples were mixed vigorously and incubated at ambient temperature overnight before counting with a PerkinElmer Life Sciences Tri-Carb 2800TR instrument. The measured disintegrations per minute (dpm) for each excised band was normalized to the optical density (OD × mm^2^) of the protein band to correct for the amount of protein applied for scintillation counting and control for unwanted variation. Dpm values not normalized to the optical density are shown in Tables S1 and Table S2. Radioactivity within the SDS-PAGE gel not caused by the photoaffinity radiolabeling experiment was corrected by substrating a background control of an excised 75-kDa protein ladder band for each gel as well as the associated non-UV-irradiated protein bands for kinesin or tubulin samples. Non-UV-irradiated protein samples displayed <5% of the measured radioactivity relative to UV-irradiated samples. Radiolabeling experiments were conducted in three technical replicates to insure the reliablilty of single values and are represented as the mean ± S.E.

### Photoaffinity labeling for microsequencing

Reaction samples were prepared similar to those mentioned above. Briefly, samples were prepared with final concentrations of 10 μm tubulin, 5 μm kinesin dimer in ATPase buffer containing 1 mm DTT, 40 μm paclitaxel, 10 μm AziP*m*, and <0.01% DMSO vehicle with or without 1 mm Mg-AMPPNP. Samples were then exposed to UV irradiation, and 20 μg of protein was precipitated using 4 volumes of chilled acetone, and proteins were precipitated overnight at −20 °C. Protein was pelleted for 20 min at 16,000 × *g* at 4 °C and then gently washed twice with 300 μl of chilled acetone. Protein pellets were resuspended in 20 μl of 0.2% ProteaseMax^TM^ Surfactant Enhancer (Promega; w/v%) in 50 mm NH_4_HCO_3_. Samples were then diluted with 74 μl of 50 mm NH_4_HCO_3_ prior to the addition of 1 μl of 0.5 m DTT and incubation at 56 °C for 20 min. Protein was then alkylated with the addition of 2.7 μl of 0.55 m iodoacetamide and incubation in the dark at room temperature for 20 min. Subsequently, 1 μl of 0.1 m CaCl_2_ and 1 μl of 1% ProteaseMax^TM^ Surfactant Enhancer (w/v%) was added, and proteins were digested overnight at 37 °C using sequencing grade trypsin (Promega) at ∼1:20 protease:protein (w/w) ratio. To stop digestion, TFA was added to 0.5% (v/v%) and samples were incubated at room temperature for 10 min. Samples were snap frozen with dry ice and stored at −80 °C until further processing. Insoluble debris was removed by centrifugation at 16,000 × *g* and the soluble peptide digest was desalted using C18 stage tips prepared in-house. The final elution was dried by SpeedVac and prior to MS analysis was resuspended in 0.1% formic acid (v/v %).

### Mass spectrometry

Desalted peptides were analyzed on an Orbitrap Elite^TM^ Hybrid Ion Trap-Orbitrap Mass Spectrometer (MS) coupled to an Easy-nanoLC 1000 system with a flow rate of 300 nl/min. Peptides were eluted with 100 min with linear gradients from 2 to 35% acetonitrile (ACN) (85 min), from 35 to 85% ACN (5 min), and finally 85% (10 min) ACN in 0.1% formic acid (v/v %). Data-dependent acquisition mode was applied with a dynamic exclusion of 45 s, in every 3-s cycle, one full MS scan was collected with a scan range of 350 to 1500 *m*/*z*, a resolution of 60,000 and the maximum injection time was 50 ms and automatic gain control of 500,000. The MS2 scans were followed from the most intense parent ions. Ions were filtered with charge 2–5 with an isolation window of 1.5 *m*/*z* in quadruple isolation mode. Ions were fragmented using collision-induced dissociation with collision energy of 35%. Ion trap detection was used with normal scan range mode and rapid ion trap scan rate. Automatic gain was set to be 10,000 with a maximal injection time of 100 ms.

### Mass spectrometry analysis

Spectral analysis was conducted using Thermo Proteome Discoverer 2.0 (Thermo Scientific) and the Mascot Daemon search engine using a customized database containing kinesin sequences supplied for heterologous expression or the bovine proteome UniProt database for WT α- and β-tubulins (UniProtKB ID P81947, Q2T9S0, Q3ZBU7, Q3MHM5, P81948, Q32KN8, Q2HJ86, Q3ZCJ7, Q6B856, Q2KJD0, Q2HJ81, and Q2HJB8). All analyses included dynamic oxidation of methionine (+15.9949 *m*/*z*) and static alkylation of cysteine (+57.0215 *m*/*z*; iodoacetamide alkylation). Photolabeled samples were run with the additional dynamic AziP*m* modification (+216.1996 *m*/*z*). A mass variation tolerance of 10 ppm for MS and 0.8 Da for MS/MS were used. Searches allowed for up to 3 missed trypsin cleavages with a false discovery rate of 0.01%. Samples were conducted in triplicate, and samples containing no photoaffinity ligand were treated similarly to control for false-positive detection of photoaffinity ligand modifications.

### Binding site prediction and docking

The X-ray crystal structures of kinesin motor domain without nucleotide (PDB ID 4LNU) (29) or with ADP-AlF_4_^−^ (PDB ID 4HNA) (30) in complex with tubulin were used for the propofol-binding site prediction and docking experiments. PyMOL (60) was used to remove the co-crystalized DARRPIN protein, ions, and crystallographic ligands as well as to mutate amino acid residues to align within with the K439 sequence. Prediction of the active site residues within the kinesin motor domain was analyzed using the CASTp web software (http://cast.engr.uic.edu)^3^ (45) with a probe radius of 1 Å. Pockets with the greatest number of photolabeled residue sites lining the cavity and predicted have a sufficient volume (>200 Å^3^) were used as the basis for docking simulations using AutoDock Vina (46). Prior to docking, the structures were prepared using AutoDock Tools4 (61) with the addition of hydrogens and Kollman charges, and the merging of nonpolar hydrogens. Flexible residues were assigned based off the amino acid residues detected by CASTp that formed the predicted pocket and were defined using AutoDock Tools4. Molecular coordinates for propofol were downloaded from the ZINC small molecule library (62) using provided physical representations. The molecular coordinates for AziP*m* were generated using MarvinSketch version 16.3.28.0 and AutoDock Tools4 to generate Gasteiger charges and to merge nonpolar hydrogens. The maximum torsions were allowed for propofol and AziP*m*, respectively (*i.e.* ligands were fully flexible). Separate docking simulations using AutoDock Vina (46) were performed similar to as previously reported (44, 63) of the entire pocket predicted by CASTp. The grid box dimensions were 24 × 26 × 20 for the no nucleotide and 28 × 26 × 26 for the ADP-AlF_4_^−^ bound kinesin in complex with tubulin at 1-Å resolution. Images were prepared using PyMOL (60).

### Statistics

GraphPad Prism 7.0 was used for preparation of graphs and statistical analysis unless otherwise noted. All *p* values are reported in the tables and figure legends, as appropriate.

## Author contributions

K. A. W., S. P. G., and R. G. E. conceptualization; K. A. W., S. G.-L., B. M. B., and N. V. B. data curation; K. A. W., S. G.-L., B. M. B., and S. P. G. formal analysis; K. A. W., S. G.-L., B. M. B., S. P. G., and R. G. E. investigation; K. A. W., S. G.-L., B. M. B., N. V. B., and R. G. E. methodology; K. A. W. and R. G. E. writing-original draft; K. A. W., S. G.-L., B. M. B., N. V. B., W. P. D., B. A. G., S. P. G., and R. G. E. writing-review and editing; B. M. B. validation; W. P. D., B. A. G., S. P. G., and R. G. E. resources; W. P. D., B. A. G., S. P. G., and R. G. E. funding acquisition; B. A. G. software; S. P. G. and R. G. E. project administration; R. G. E. supervision.

## Supplementary Material

Supporting Information

## Footnotes

3 Please note that the JBC is not responsible for the long-term archiving and maintenance of this site or any other third party hosted site.

## Footnotes

2 The abbreviations used are:

AziP*m*: *meta*-azipropofol
ACN: acetonitrile
AMPPNP: adenosine 5′-(β,γ-imino)-triphosphate
ADP-AlF_4_^−^: adenosine diphosphate-aluminum fluoride
CASTp: Computed Atlas of Surface Topography of proteins
GABA_A_: γ-aminobutyric acid type A
IPTG: isopropyl β-d-1-thiogalactopyranoside
LB: lysogeny broth
Qdot: quantum-dot
SHD: synthetic heterodimerization helix
TIVA: total intravenous administration
TIRF: total internal reflection fluorescence
TEV: tobacco etch virus.

## References

[1] Bentley M., and Banker G. (2016) The cellular mechanisms that maintain neuronal polarity. Nat. Rev. Neurosci. 17, 611–622 10.1038/nrn.2016.100 27511065

[2] Hirokawa N., Niwa S., and Tanaka Y. (2010) Molecular motors in neurons: transport mechanisms and roles in brain function, development, and disease. Neuron 68, 610–638 10.1016/j.neuron.2010.09.039 21092854

[3] Maday S., Twelvetrees A. E., Moughamian A. J., and Holzbaur E. L. (2014) Axonal transport: cargo-specific mechanisms of motility and regulation. Neuron 84, 292–309 10.1016/j.neuron.2014.10.019 25374356 PMC4269290

[4] Hirokawa N., Noda Y., Tanaka Y., and Niwa S. (2009) Kinesin superfamily motor proteins and intracellular transport. Nat. Rev. Mol. Cell Biol. 10, 682–696 10.1038/nrm2774 19773780

[5] Miki H., Setou M., Kaneshiro K., and Hirokawa N. (2001) All kinesin superfamily protein, KIF, genes in mouse and human. Proc. Natl. Acad. Sci. U.S.A. 98, 7004–7011 10.1073/pnas.111145398 11416179 PMC34614

[6] Viancour T. A., and Kreiter N. A. (1993) Vesicular fast axonal transport rates in young and old rat axons. Brain Res. 628, 209–217 10.1016/0006-8993(93)90957-O 8313149

[7] Kaether C., Skehel P., and Dotti C. G. (2000) Axonal membrane proteins are transported in distinct carriers: a two-color video microscopy study in cultured hippocampal neurons. Mol. Biol. Cell 11, 1213–1224 10.1091/mbc.11.4.1213 10749925 PMC14842

[8] Lasek R. J., Garner J. A., and Brady S. T. (1984) Axonal transport of the cytoplasmic matrix. J. Cell Biol. 99, 212s–221s 10.1083/jcb.99.1.212s 6378920 PMC2275578

[9] Paschal B. M., and Vallee R. B. (1987) Retrograde transport by the microtubule-associated protein MAP 1C. Nature 330, 181–183 10.1038/330181a0 3670402

[10] Paschal B. M., Shpetner H. S., and Vallee R. B. (1987) MAP 1C is a microtubule-activated ATPase which translocates microtubules *in vitro* and has dynein-like properties. J. Cell Biol. 105, 1273–1282 10.1083/jcb.105.3.1273 2958482 PMC2114794

[11] Fu M., and Holzbaur E. L. (2014) Integrated regulation of motor-driven organelle transport by scaffolding proteins. Trends Cell Biol. 24, 564–574 10.1016/j.tcb.2014.05.002 24953741 PMC4177981

[12] Verhey K. J., Kaul N., and Soppina V. (2011) Kinesin assembly and movement in cells. Annu. Rev. Biophys. 40, 267–288 10.1146/annurev-biophys-042910-155310 21332353

[13] Kozielski F., Sack S., Marx A., Thormählen M., Schönbrunn E., Biou V., Thompson A., Mandelkow E. M., and Mandelkow E. (1997) The crystal structure of dimeric kinesin and implications for microtubule-dependent motility. Cell 91, 985–994 10.1016/S0092-8674(00)80489-4 9428521

[14] Kull F. J., Sablin E. P., Lau R., Fletterick R. J., and Vale R. D. (1996) Crystal structure of the kinesin motor domain reveals a structural similarity to myosin. Nature 380, 550–555 10.1038/380550a0 8606779 PMC2851642

[15] Aizawa H., Sekine Y., Takemura R., Zhang Z., Nangaku M., and Hirokawa N. (1992) Kinesin family in murine central nervous system. J. Cell Biol. 119, 1287–1296 10.1083/jcb.119.5.1287 1447303 PMC2289715

[16] Muresan V., Abramson T., Lyass A., Winter D., Porro E., Hong F., Chamberlin N. L., and Schnapp B. J. (1998) KIF3C and KIF3A form a novel neuronal heteromeric kinesin that associates with membrane vesicles. Mol. Biol. Cell 9, 637–652 10.1091/mbc.9.3.637 9487132 PMC25292

[17] Sardella M., Navone F., Rocchi M., Rubartelli A., Viggiano L., Vignali G., Consalez G. G., Sitia R., and Cabibbo A. (1998) KIF3C, a novel member of the kinesin superfamily: sequence, expression, and mapping to human chromosome 2 at 2p23. Genomics 47, 405–408 10.1006/geno.1997.5123 9480755

[18] Yamazaki H., Nakata T., Okada Y., and Hirokawa N. (1995) KIF3A/B: a heterodimeric kinesin superfamily protein that works as a microtubule plus end-directed motor for membrane organelle transport. J. Cell Biol. 130, 1387–1399 10.1083/jcb.130.6.1387 7559760 PMC2120571

[19] Yang Z., and Goldstein L. S. (1998) Characterization of the KIF3C neural kinesin-like motor from mouse. Mol. Biol. Cell 9, 249–261 10.1091/mbc.9.2.249 9450952 PMC25248

[20] Hua W., Young E. C., Fleming M. L., and Gelles J. (1997) Coupling of kinesin steps to ATP hydrolysis. Nature 388, 390–393 10.1038/41118 9237758

[21] Schnitzer M. J., and Block S. M. (1997) Kinesin hydrolyses one ATP per 8-nm step. Nature 388, 386–390 10.1038/41111 9237757

[22] Svoboda K., Schmidt C. F., Schnapp B. J., and Block S. M. (1993) Direct observation of kinesin stepping by optical trapping interferometry. Nature 365, 721–727 10.1038/365721a0 8413650

[23] Asbury C. L., Fehr A. N., and Block S. M. (2003) Kinesin moves by an asymmetric hand-over-hand mechanism. Science 302, 2130–2134 10.1126/science.1092985 14657506 PMC1523256

[24] Kaseda K., Higuchi H., and Hirose K. (2003) Alternate fast and slow stepping of a heterodimeric kinesin molecule. Nat. Cell Biol. 5, 1079–1082 10.1038/ncb1067 14634664

[25] Yildiz A., Tomishige M., Vale R. D., and Selvin P. R. (2004) Kinesin walks hand-over-hand. Science 303, 676–678 10.1126/science.1093753 14684828

[26] Bensel B. M., Guzik-Lendrum S., Masucci E. M., Woll K. A., Eckenhoff R. G., and Gilbert S. P. (2017) Common general anesthetic propofol impairs kinesin processivity. Proc. Natl. Acad. Sci. U.S.A. 144, E4281–E4287 28484025 10.1073/pnas.1701482114 PMC5448234

[27] Friedman E. B., Sun Y., Moore J. T., Hung H.-T., Meng Q. C., Perera P., Joiner W. J., Thomas S. A., Eckenhoff R. G., Sehgal A., and Kelz M. B. (2010) A conserved behavioral state barrier impedes transitions between anesthetic-induced unconsciousness and wakefulness: evidence for neural inertia. PLoS ONE 5, e11903 10.1371/journal.pone.0011903 20689589 PMC2912772

[28] Hall M. A., Xi J., Lor C., Dai S., Pearce R., Dailey W. P., and Eckenhoff R. G. (2010) m-Azipropofol (AziPm) a photoactive analogue of the intravenous general anesthetic propofol. J. Med. Chem. 53, 5667–5675 10.1021/jm1004072 20597506 PMC2917171

[29] Cao L., Wang W., Jiang Q., Wang C., Knossow M., and Gigant B. (2014) The structure of apo-kinesin bound to tubulin links the nucleotide cycle to movement. Nat. Commun. 5, 5364 10.1038/ncomms6364 25395082

[30] Gigant B., Wang W., Dreier B., Jiang Q., Pecqueur L., Plückthun A., Wang C., and Knossow M. (2013) Structure of a kinesin-tubulin complex and implications for kinesin motility. Nat. Struct. Mol. Biol. 20, 1001–1007 10.1038/nsmb.2624 23872990

[31] Liu D., Liu X., Shang Z., and Sindelar C. V. (2017) Structural basis of cooperativity in kinesin revealed by 3D reconstruction of a two-head-bound state on microtubules. Elife 10.7554/eLife.24490 PMC5459574 28504639

[32] Rice S., Lin A. W., Safer D., Hart C. L., Naber N., Carragher B. O., Cain S. M., Pechatnikova E., Wilson-Kubalek E. M., Whittaker M., Pate E., Cooke R., Taylor E. W., Milligan R. A., and Vale R. D. (1999) A structural change in the kinesin motor protein that drives motility. Nature 402, 778–784 10.1038/45483 10617199

[33] Shang Z., Zhou K., Xu C., Csencsits R., Cochran J.C, and Sindelar C. V. (2014) High-resolution structures of kinesin on microtubules provide a basis for nucleotide-gated force-generation. Elife 3, e04686 25415053 10.7554/eLife.04686 PMC4383081

[34] Hirokawa N., Sato-Yoshitake R., Kobayashi N., Pfister K. K., Bloom G. S., and Brady S. T. (1991) Kinesin associates with anterogradely transported membranous organelles *in vivo*. J. Cell Biol. 114, 295–302 10.1083/jcb.114.2.295 1712789 PMC2289077

[35] Takeda S., Yamazaki H., Seog D. H., Kanai Y., Terada S., and Hirokawa N. (2000) Kinesin superfamily protein 3 (KIF3) motor transports fodrin-associating vesicles important for neurite building. J. Cell Biol. 148, 1255–1265 10.1083/jcb.148.6.1255 10725338 PMC2174314

[36] Castle M. J., Perlson E., Holzbaur E. L., and Wolfe J. H. (2014) Long-distance axonal transport of AAV9 is driven by dynein and kinesin-2 and is trafficked in a highly motile Rab7-positive compartment. Mol. Ther. 22, 554–566 10.1038/mt.2013.237 24100640 PMC3944332

[37] Hendricks A. G., Perlson E., Ross J. L., Schroeder H. W. 3rd, Tokito M., and Holzbaur E. L. (2010) Motor coordination via a tug-of-war mechanism drives bidirectional vesicle transport. Curr. Biol. 20, 697–702 10.1016/j.cub.2010.02.058 20399099 PMC2908734

[38] Block S. M., Goldstein L. S., and Schnapp B. J. (1990) Bead movement by single kinesin molecules studied with optical tweezers. Nature 348, 348–352 10.1038/348348a0 2174512

[39] Lasek R. J., and Brady S. T. (1985) Attachment of transported vesicles to microtubules in axoplasm is facilitated by AMP-PNP. Nature 316, 645–647 10.1038/316645a0 4033761

[40] Vale R. D., Reese T. S., and Sheetz M. P. (1985) Identification of a novel force-generating protein, kinesin, involved in microtubule-based motility. Cell 42, 39–50 10.1016/S0092-8674(85)80099-4 3926325 PMC2851632

[41] Woll K. A., Dailey W. P., Brannigan G., and Eckenhoff R. G. (2016) Shedding light on anesthetic mechanisms: application of photoaffinity ligands. Anesth. Analg. 123, 1253–1262 10.1213/ANE.0000000000001365 27464974 PMC5072990

[42] Bayley H. (1983) Photogenerated Reagents in Biochemistry and Molecular Biology, Laboratory Techniques in Biochemistry and Molecular Biology, Elsevier, New York 10.1016/S0075-7535(08)70436-4

[43] Weiser B. P., Woll K. A., Dailey W. P., and Eckenhoff R. G. (2014) Mechanisms revealed through general anesthetic photolabeling. Curr. Anesth. Rep. 4, 57–66 10.1007/s40140-013-0040-7 PMC3927792 24563623

[44] Jayakar S. S., Zhou X., Chiara D. C., Dostalova Z., Savechenkov P. Y., Bruzik K. S., Dailey W. P., Miller K. W., Eckenhoff R. G., and Cohen J. B. (2014) Multiple propofol binding sites in a γ-aminobutyric acid type A receptor (GABAAR) identified using a photoreactive propofol analog. J. Biol. Chem. 289, 27456–27468 10.1074/jbc.M114.581728 25086038 PMC4183786

[45] Dundas J., Ouyang Z., Tseng J., Binkowski A., Turpaz Y., and Liang J. (2006) CASTp: computed atlas of surface topography of proteins with structural and topographical mapping of functionally annotated residues. Nucleic Acids Res. 34, W116–8 10.1093/nar/gkl282 16844972 PMC1538779

[46] Trott O., and Olson A. J. (2010) AutoDock Vina: improving the speed and accuracy of docking with a new scoring function, efficient optimization, and multithreading. J. Comput. Chem. 31, 455–461 19499576 10.1002/jcc.21334 PMC3041641

[47] Tischfield M. A., Baris H. N., Wu C., Rudolph G., Van Maldergem L., He W., Chan W.-M., Andrews C., Demer J. L., Robertson R. L., Mackey D. A., Ruddle J. B., Bird T. D., Gottlob I., Pieh C., et al (2010) Human TUBB3 mutations perturb microtubule dynamics, kinesin interactions, and axon guidance. Cell 140, 74–87 10.1016/j.cell.2009.12.011 20074521 PMC3164117

[48] Tischfield M. A., Cederquist G. Y., Gupta M. L. Jr., and Engle E. C. (2011) Phenotypic spectrum of the tubulin-related disorders and functional implications of disease-causing mutations. Curr. Opin. Genet. Dev. 21, 286–294 10.1016/j.gde.2011.01.003 21292473 PMC3100401

[49] Niwa S., Takahashi H., and Hirokawa N. (2013) β-Tubulin mutations that cause severe neuropathies disrupt axonal transport. EMBO J. 32, 1352–1364 10.1038/emboj.2013.59 23503589 PMC3655465

[50] Minoura I., Takazaki H., Ayukawa R., Saruta C., Hachikubo Y., Uchimura S., Hida T., Kamiguchi H., Shimogori T., and Muto E. (2016) Reversal of axonal growth defects in an extraocular fibrosis model by engineering the kinesin-microtubule interface. Nat. Commun. 7, 10058 10.1038/ncomms10058 26775887 PMC4735607

[51] Ulaganathan V., Talapatra S. K., Rath O., Pannifer A., Hackney D. D., and Kozielski F. (2013) Structural insights into a unique inhibitor binding pocket in kinesin spindle protein. J. Am. Chem. Soc. 135, 2263–2272 10.1021/ja310377d 23305346

[52] Yan Y., Sardana V., Xu B., Homnick C., Halczenko W., Buser C. A., Schaber M., Hartman G. D., Huber H. E., and Kuo L. C. (2004) Inhibition of a mitotic motor protein: where, how, and conformational consequences. J. Mol. Biol. 335, 547–554 10.1016/j.jmb.2003.10.074 14672662

[53] Wood K. W., Lad L., Luo L., Qian X., Knight S. D., Nevins N., Brejc K., Sutton D., Gilmartin A. G., Chua P. R., Desai R., Schauer S. P., McNulty D. E., Annan R. S., Belmont L. D., et al (2010) Antitumor activity of an allosteric inhibitor of centromere-associated protein-E. Proc. Natl. Acad. Sci. U.S.A. 107, 5839–5844 10.1073/pnas.0915068107 20167803 PMC2851928

[54] Olsen R. W., and Li G. D. (2011) GABA(A) receptors as molecular targets of general anesthetics: identification of binding sites provides clues to allosteric modulation. Can. J. Anaesth. 58, 206–215 10.1007/s12630-010-9429-7 21194017 PMC3033524

[55] Bryson H. M., Fulton B. R., and Faulds D. (1995) Propofol- An update of its use in anesthesia and counsious sedation. Drugs 50, 513–559 10.2165/00003495-199550030-00008 8521772

[56] Leite L. F., Gomez R. S., Fonseca M. de C., Gomez M. V., and Guatimosim C. (2011) Effect of intravenous anesthetic propofol on synaptic vesicle exocytosis at the frog neuromuscular junction. Acta Pharmacol. Sin. 32, 31–37 10.1038/aps.2010.175 21113178 PMC4003312

[57] Mintz C. D., Smith S. C., Barrett K. M., and Benson D. L. (2012) Anesthetics interfere with the polarization of developing cortical neurons. J. Neurosurg. Anesth. 24, 368–375 10.1097/ANA.0b013e31826a03a6 PMC3479440 23085784

[58] Albracht C. D., Rank K. C., Obrzut S., Rayment I., and Gilbert S. P. (2014) Kinesin-2 KIF3AB exhibits novel ATPase characteristics. J. Biol. Chem. 289, 27836–27848 10.1074/jbc.M114.583914 25122755 PMC4183818

[59] Guzik-Lendrum S., Rank K. C., Bensel B. M., Taylor K. C., Rayment I., and Gilbert S. P. (2015) Kinesin-2 KIF3AC and KIF3AB can drive long-range transport along microtubules. Biophys. J. 109, 1472–1482 10.1016/j.bpj.2015.08.004 26445448 PMC4601047

[60] Schrödinger LLC. (2015) The PyMOL Molecular Graphics System, version 1.8, Schroedinger, LLC, New York

[61] Morris G. M., Huey R., Lindstrom W., Sanner M. F., Belew R. K., Goodsell D. S., and Olson A. J. (2009) AutoDock4 and AutoDockTools4: automated docking with selective receptor flexibility. J. Comput. Chem. 30, 2785–2791 10.1002/jcc.21256 19399780 PMC2760638

[62] Irwin J. J., Sterling T., Mysinger M. M., Bolstad E. S., and Coleman R. G. (2012) ZINC: a free tool to discover chemistry for biology. J. Chem. Inf. Model 52, 1757–1768 10.1021/ci3001277 22587354 PMC3402020

[63] Woll K. A., Peng W., Liang Q., Zhi L., Jacobs J. A., Maciunas L., Bhanu N., Garcia B. A., Covarrubias M., Loll P. J., Dailey W. P., and Eckenhoff R. G. (2017) Photoaffinity ligand for the inhalational anesthetic sevoflurane allows mechanistic insight into potassium channel modulation. ACS Chem. Biol. 12, 1353–1362 10.1021/acschembio.7b00222 28333442

