# Supplementary material for: An allosteric propofol-binding site in kinesin disrupts kinesin-mediated processive movement on microtubules
Source: J Biol Chem. 2018 May 29;293(29):11283–95. doi: 10.1074/jbc.RA118.002182 (PMC6065180; doi:10.1074/jbc.RA118.002182)
Supplement: Supporting Information [file supp_293_29_11283__index.html]

An allosteric propofol-binding site in kinesin disrupts kinesin-mediated processive movement on microtubules — Identification of inhibitory propofol site within kinesins — An allosteric propofol-binding site in kinesin disrupts kinesin-mediated processive movement on microtubules — Identification of inhibitory propofol site within kinesins — Supporting Information 

# An allosteric propofol-binding site in kinesin disrupts kinesin-mediated processive movement on microtubules

## Supporting Information

- Supporting Information - Supporting Information, Figures S1-S14 and Tables S1-S2
