# Supplementary material for: An allosteric propofol-binding site in kinesin disrupts kinesin-mediated processive movement on microtubules
Source: J Biol Chem. 2018 May 29;293(29):11283–95. doi: 10.1074/jbc.RA118.002182 (PMC6065180; doi:10.1074/jbc.RA118.002182)
Supplement: Supporting Information [file supp_RA118.002182_135668_2_supp_143842_p996vw.pdf]

SUPPORTING INFORMATION

Novel allosteric binding site for the selective disruption of kinesin processive movement by propofol

Kellie A. Woll, Stephanie Guzik-Lendrum, Brandon M. Bensel, Natarajan V. Bhanu, Benjamin A. Garcia, William P. Dailey, Susan P. Gilbert, Roderic G. Eckenhoff *

Table of contents

Pages.

2	**Table S1**
2	**Table S2**
3	**Figure S1**
4	**Figure S2**
5	**Figure S3**
6	**Figure S4**
7	**Figure S5**
8	**Figure S6**
9	**Figure S7**
10	**Figure S8**
11	**Figure S9**
12	**Figure S10**
13	**Figure S11**
14-15	**Figure S12**
16	**Figure S13**
17	**Figure S14**
18	**References**

Table S1. *[^{3}H]AziPm photoaffinity radiolabeling of kinesin and microtubule•kinesin (MT•Kinesin) complexes with and without AMPPNP and unlabeled propofol*

	Kinesin (dpm)	MT•Kinesin (∅) (dpm)		MT•Kinesin + AMPPNP (dpm)	
		- propofol	+ propofol	- propofol	+ propofol
K439	327.9 ± 71.30	582.4 ± 143.80	481.7 ± 60.95	1242.5 ± 124.91	599.5 ± 121.4
KIF3AB	248.2 ± 37.62	320.8 ± 69.22	332.8 ± 61.60	916.8 ± 163.34	391.1 ± 90.39
KIF3AC	233.9 ± 35.56	373.7 ± 68.39	391.64± 88.96	933.0 ± 131.04	371.7 ± 63.88

The MT•Kinesin complex (10 µM MTs + 5 µM kinesin dimer) was preformed in the absence of nucleotide (∅) or in the presence of 1 mM AMPPNP with or without 400 µM propofol. Values are shown as mean ± SEM of three experimental replicates.

Table S2. *[^{3}H]AziPm photoaffinity radiolabeling of microtubules and microtubule•kinesin (MT•Kinesin) complexes with and without AMPPNP and unlabeled propofol*

	Microtubule (dpm)	MT•Kinesin (∅) (dpm)		MT•Kinesin + AMPPNP (dpm)	
		- propofol	+ propofol	- propofol	+ propofol
Tubulin (K439)	545.2 ± 128.28	249.5 ± 99.15	623.0 ± 212.78	844.4 ± 372.46	1022.9 ± 265.94
Tubulin (KIF3AB)	491.4 ± 194.07	295.8 ± 53.69	736.7 ± 132.37	1434.7 ± 221.02	926.67 ± 253.27
Tubulin (KIF3AC)	110.3 ± 35.40	199.4 ± 47.01	506.4 ± 127.34	692.0 ± 92.15	639.4 ± 114.17

The MT•Kinesin complex (10 µM MTs + 5 µM kinesin dimer) was preformed with or without (∅) 1 mM AMPPNP and with (+ propofol) or without (- propofol) 400 µM unlabeled propofol. Values are shown as Mean ± SEM of three experimental replicates

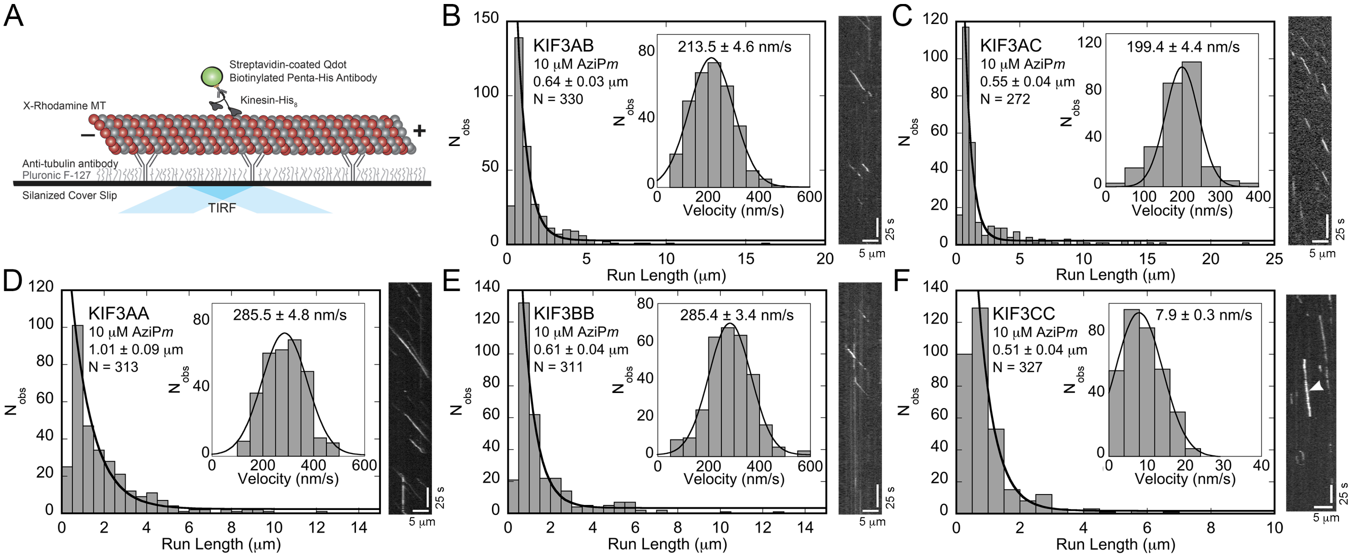

G

Motor	Run Length (μm) + 5% DMSO	Run Length (μm) + 10 μM AziP*m*	Velocity (nm/s) + 5% DMSO	Velocity (nm/s) + 10 μM AziP*m*
K439	1.34 ± 0.10	0.67 ± 0.06	320.9 ± 5.2	313.6 ± 2.0
KIF3AB	1.61 ± 0.33[a]	0.64 ± 0.03	207.8 ± 2.3[a]	213.5 ± 4.6
KIF3AC	1.16 ± 0.05[a]	0.55 ± 0.04	182.1 ± 5.4[a]	199.4 ± 4.4
KIF3AA	1.05 ± 0.06[a]	1.01 ± 0.09	286.1 ± 7.7[a]	285.5 ± 4.8
KIF3BB	1.29 ± 0.17[a]	0.61 ± 0.04	281.5 ±11.0[a]	285.4 ± 3.4
KIF3CC	0.79 ± 0.07[a]	0.51 ± 0.04	6.9 ± 0.4[a]	7.9 ± 0.3

Figure S1. Like propofol, AziP*m* shortens the run length potential of kinesin-2 heterodimeric and homodimeric motors except for KIF3AA. (A) Illustration of single molecule method for analysis of individual Qdot-labeled kinesin motors moving along X-rhodamine labeled microtubule tracks. (B – F) Histograms of single molecule run length and velocity (*inset*) data and representative kymographs in the presence of 10 μM AziP*m* for kinesin-2 motors (B) KIF3AB, (C) KIF3AC, and engineered homodimers (D) KIF3AA, (E) KIF3BB and (F) KIF3CC. The KIF3CC behavior differs greatly from other kinesin-2s, as illustrated by a representative run highlighted in its kymograph (*arrowhead*) with an exceedingly slow velocity of 3.7 nm/s and a run length (the x-axis dimension of the run) of 1.4 μm. All experiments were conducted in the presence of 1 mM MgATP at 25 °C, with values reported as mean ± SEM. Kymograph scale bars: 5 μm along the x-axis, 25 s along the y-axis. (G) Compiled single-molecule motility data for the kinesin-1 and kinesin-2 motors from Figure 2 and Figure S2. Statistical comparison of AziP*m* datasets with DMSO control values showed significant differences in run lengths for KIF3AB, KIF3AC, KIF3BB and KIF3CC ($p < 0.002$). In contrast, KIF3AA showed no statistical significance between AziP*m* and a DMSO control ($p > 0.3$). Mean velocities were not statistically significant between AziP*m* and DMSO control values for kinesin-2 motors, with the exception of KIF3AC which showed moderate significance for an increased velocity between the two conditions ($p > 0.02$). All values are reported mean ± SEM. [a], DMSO control data for comparison were previously reported in Bensel et al 2017 (1).

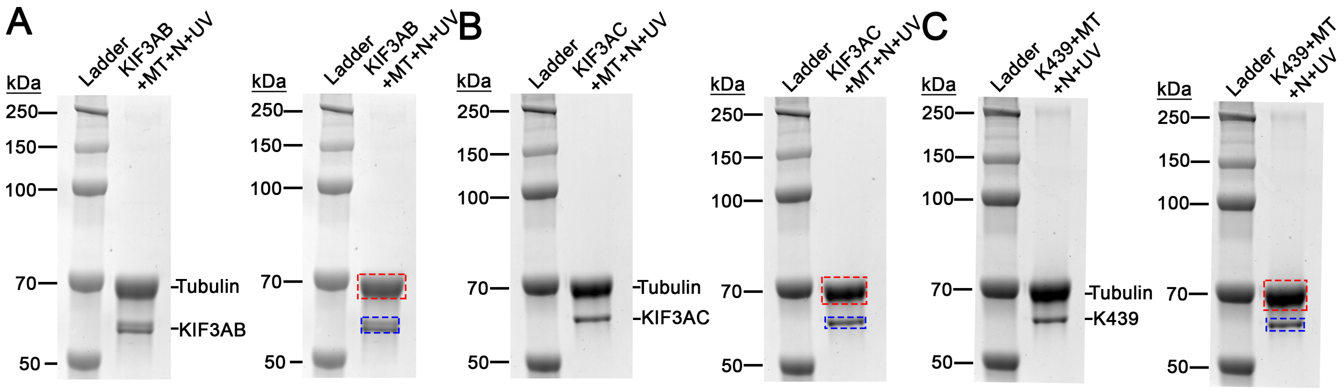

Figure S2. Representative Coomassie Blue G250 stained SDS-PAGE gels of irradiated KIF3AB (A), KIF3AC (B) or K439 (C) in the presence of microtubules (MT) and AMP-PNP. Left gel image displays the protein components labeled accordingly with adjasent SDS-PAGE protein ladder. Right gel image shows the approximate excised bands of the gels for tubulin (red) and kinesin dimer (blue) outlined in a dashed box.

A M**ADLAECNIK VMCRFRPLNE SEVNRGDKYI AKFQGEDTVV IASKPYAFDR VFQSSTSQEQ VYNDCAKKIV KDVLEGYNGT**
IFAYGQTSSG KTHTMEGKLH DPEGMGIIPR IVQDIFNYIY SMDENLEFHI KVSYFEIYLD KIRDLLDVSK TNLSVHEDKN
RVPYVKGCTE R**FVCSPDEVM DTIDEGKSNR HVAVTNMNEH SSRSHSIFLI NVKQENTQTE QKLSGKLYLV DLAGSEKVSK**
TGAEGAVLDE AKNINKSLSA LGNVISALAE GSTYVPYRDS KMTRILQDSL GGNCRTTIVI CCSPSSYNES ETKSTLLFGQ
RAKTIK**NTVC VNVELTAEQW KK**KYEKEKEK NKILR**NTIQW LENELNRWRN GETVPIDEQF DKEKANLEAF TVDKDITLTN**
DKPATAIGVI GNFTDAERRK CEEEIAKLYK QLDDKDEEID FYFGKLRNIE LICQENEGEN DPVLQRIVDI LYATDETTSE
NLYFQGASHH HHHHHH

B M**ADLAECNIK VMCRFRPLNE SEVNRGDKYI AKFQGEDTVV IASKPYAFDR VFQSSTSQEQ VYNDCAKKIV KDVLEGYNGT**
IFAYGQTSSG KTHTMEGKLH DPEGMGIIPR IVQDIFNYIY SMDENLEFHI KVSYFEIYLD KIRDLLDVSK TNLSVHEDKN
RVPYVKGCTE R**FVCSPDEVM DTIDEGKSNR HVAVTNMNEH SSRSHSIFLI NVKQENTQTE QKLSGKLYLV DLAGSEKVSK**
TGAEGAVLDE AKNINKSLSA LGNVISALAE GSTYVPYRDS KMTRILQDSL GGNCRTTIVI CCSPSSYNES ETKSTLLFGQ
RAKTIK**NTVC VNVELTAEQW KK**KYEKEKEK NK**ILRNTIQW LENELNRWRN GETVPIDEQF DKEKANLEAF TVDKDITLTN**
DKPATAIGVI GNFTDAERRK CEEEIAKLYK QLDDKDEEID FYFGKLRNIE LICQENEGEN DPVLQRIVDI LYATDETTSE
NLYFQGASHH HHHHHH

Figure S3. *Coverage maps for K439 mass spectrometry analysis*. Sequences of the K439 with high confidence coverage in the mass spectrometry analysis denoted as black residue codes in systems with (A; 95.2% coverage) and without (B; 95.8% coverage) AMP-PNP.

A MPINKSEKPE SCDNVKVVVR **CRPLNER**EKS MCYR**QAVSVD** **EMRGTITVHK** **TDSSNEPPKT** **FTFDTVFGPE** **SKQLDVYNLT**
ARPIIDSVLE **GYNGTIFAYG** **QTGTGKTFTM** **EGVRAVPGLR** **GVIPNSFAHI** **FGHIAK**AEGD TRFLVR**VSYL** **EIYNEEVRDL**
LGKDQTQRLE **VKERPDVGVY** **IKDLSAYVVN** **NADDMDRIMT** **LGHK**NR**SVGA** **TNMNEHSSRS** **HAIFTITIEC** **SEKGVDGNMH**
VRMGKLHLVD **LAGSERQAKT** **GATGQRLKEA** **TKINLSLSTL** **GNVISALVDG** **KSTHVPYR**NS KLTR**LLQDSL** **GGNSKTMMCA**
NIGPADYNYD **ETISTLR**YAN RAKNIKNKAR **INEDPKDALL** **RQFQKEIEEL** **KKKLEELEKE** **IAALEKEIAA** **LEKTTSENLY**
FQGASNWSHP **QFEK**

B M**PINKSEKPE** **SCDNVKVVVR** **CRPLNER**EKS MCYR**QAVSVD** **EMRGTITVHK** **TDSSNEPPKT** **FTFDTVFGPE** **SKQLDVYNLT**
ARPIIDSVLE **GYNGTIFAYG** **QTGTGKTFTM** **EGVR**AVPGLR **GVIPNSFAHI** **FGHIAKAEGD** **TRFLVRVSYL** **EIYNEEVRDL**
LGKDQTQRLE **VKERPDVGVY** **IKDLSAYVVN** **NADDMDRIMT** **LGHK**NR**SVGA** **TNMNEHSSRS** **HAIFTITIEC** **SEKGVDGNMH**
VRMGK**LHLVD** **LAGSER**QAKT GATGQRLKEA TK**INLSLSTL** **GNVISALVDG** **KSTHVPYR**NS KLTR**LLQDSL** **GGNSKTMMCA**
NIGPADYNYD **ETISTLR**YAN RAKNIKNKAR **INEDPKDALL** **RQFQKEIEEL** **KKKLEELEKE** **IAALEKEIAA** **LEKTTSENLY**
FQGASNWSHP **QFEK**

C MPINK**SEKPE** **SCDNVKVVVR** **CRPLNER**EKS MCYR**QAVSVD** **EMRGTITVHK** **TDSSNEPPKT** **FTFDTVFGPE** **SKQLDVYNLT**
ARPIIDSVLE **GYNGTIFAYG** **QTGTGKTFTM** **EGVR**AVPGLR **GVIPNSFAHI** **FGHIAK**AEGD TRFLVR**VSYL** **EIYNEEVRDL**
LGKDQTQRLE VK**ERPDVGVY** **IKDLSAYVVN** **NADDMDRIMT** **LGHK**NR**SVGA** **TNMNEHSSRS** **HAIFTITIEC** **SEKGVDGNMH**
VRMGKLHLVD **LAGSERQAK**T GATGQRLKEA TK**INLSLSTL** **GNVISALVDG** **KSTHVPYR**NS KLTR**LLQDSL** **GGNSKTMMCA**
NIGPADYNYD **ETISTLR**YAN RAKNIKNKAR **INEDPKDALL** **RQFQKEIEEL** **KKKLEELEKE** **IAALEKEIAA** **LEKTTSENLY**
FQGASNWSHP **QFEK**

D MPINKSEKPE SCDNVK**VVVR** **CRPLNEREKS** **MCYRQAVSVD** **EMRGTITVHK** **TDSSNEPPKT** **FTFDTVFGPE** **SKQLDVYNLT**
ARPIIDSVLE **GYNGTIFAYG** **QTGTGKTFTM** **EGVR**AVPGLR **GVIPNSFAHI** **FGHIAKAEGD** **TRFLVRVSYL** **EIYNEEVRDL**
LGKDQTQRLE VK**ERPDVGVY** **IKDLSAYVVN** **NADDMDRIMT** **LGHK**NR**SVGA** **TNMNEHSSRS** **HAIFTITIEC** **SEKGVDGNMH**
VRMGKLHLVD **LAGSER**QAKT GATGQRLKEA TK**INLSLSTL** **GNVISALVDG** **KSTHVPYRNS** **KLTRLLQDSL** **GGNSKTMMCA**
NIGPADYNYD **ETISTLR**YAN RAKNIKNKAR **INEDPKDALL** **RQFQKEIEEL** **KKKLEELEKE** **IAALEKEIAA** **LEKTTSENLY**
FQGASNWSHP **QFEK**

Figure S4. *Coverage maps for KIF3A mass spectrometry analysis*. Sequences of the KIF3A in KIF3AB systems with high confidence coverage in the mass spectrometry analysis denoted as black residue codes in systems with (A; 82.1% coverage) and without (B; 87.0% coverage) AMP-PNP. Sequences of the KIF3A in KIF3AC systems with high confidence coverage in the mass spectrometry analysis denoted as black residue codes in systems with (C; 80.6% coverage) and without (D; 82.6% coverage) AMP-PNP.

A MSKLKSSESV RVVVRCRPMN GKEK**AASYDK** **VVDVDVKLGQ** **VSVKNPKGTS** **HEMPKTFTFD** **AVYDWNAKQF** **ELYDETFRPL**
VDSVLQGFNG **TIFAYGQTGT** **GKTYTMEGVR** **GDPEKRGVIP** **NSFDHIFTHI** **SRSQNQQYLV** **RASYLEIYQE** **EIRDLLSKDQ**
TKRLELK**ERP** **DTGVYVKDLS** **SFVTKSVKEI** **EHVMNVGNQN** **RSVGATNMNE** **HSSRSHAIFV** **ITIECSEVGL** **DGENHIR**VGK
LNLVDLAGSE **R**QAKTGAQGE RLKEATK**INL** **SLSALGNVIS** **ALVDGKSTHI** **PYRDSK**LTR**L** **LQDSLGGNAK** **TVMVANVGPA**
SYNVEETLTT **LR**YANRAKNI KNKPR**VNEDP** **KDALLREFQE** **EIARLKAQLE** **KLKEKIAALK** EKIAALK**ETT** **SENLYFQGAS**
HHHHHHHH

B MSKLKSSESV RVVVRCRPMN GK**EKAASYDK** **VVDVDVKLGQ** **VSVKNPKGTS** **HEMPKTFTFD** **AVYDWNAKQF** **ELYDETFRPL**
VDSVLQGFNG **TIFAYGQTGT** **GKTYTMEGVR** GDPEK**RGVIP** **NSFDHIFTHI** **SRSQNQQYLV** **RASYLEIYQE** **EIRDLLSK**DQ
TKRLELK**ERP** **DTGVYVKDLS** **SFVTK**SVK**EI** **EHVMNVGNQN** **RSVGATNMNE** **HSSRSHAIFV** **ITIECSEVGL** **DGENHIRVGK**
LNLVDLAGSE **R**QAKTGAQGE RLK**EATKINL** **SLSALGNVIS** **ALVDGKSTHI** **PYRDSK**LTR**L** **LQDSLGGNAK** **TVMVANVGPA**
SYNVEETLTT **LR**YANRAK**NI** **KNKPRVNEDP** **KDALLREFQE** **EIARLKAQLE** **K**LKEK**IAALK** **EKIAALKETT** **SENLYFQGAS**
HHHHHHHH

Figure S5. *Coverage maps for KIF3B mass spectrometry analysis.* Sequences of the KIF3B with high confidence coverage in the mass spectrometry analysis denoted as black residue codes in systems with (A; 82.6% coverage) and without (B; 84.3% coverage) AMP-PNP.

A MASKTKASEA LKVVARCRPL SR**KEEAAGHE** **QILTMDVKLG** **QVTLR**NPR**AA** **PGELPKTFTF** **DAVYDASSKQ** **ADLYDETVRP**
LIDSVLQGFN **GTVFAYGQTG** **TGKTYTMQGT** **WVEPELRGVI** **PNAFEHIFTH** **ISRSQNQQYL** **VRASYLEIYQ** **EEIRDLLSKE**
PGKRLELKEN **PETGVYIKDL** **SSFVTKNVKE** **IEHVMNLGNQ** **ARAVGSTHMN** **EVSSRSHAIF** **VITVECSER**G SDGQDHIR**VG**
KLNLVDLAGS **ER**QNK**AGPNA** **AGGPATQPTA** **GGGSGSGSAS** **GSASSGERPK** **EASKINLSLS** **ALGNVIAALA** **GNRSTHIPYR**
DSKLTR**LLQD** **SLGGNAKTIM** **VATLGPASHS** **YDESLSTLR**F ANRAKNIKNK PR**VNEDPKDT** **LLREFQEEIA** **RLKAQLDFYF**
GKLRNIELIC **QENEGENDPV** **LQRIVDILYA** **TDETTSENLY** **FQGASHHHHH** **HHH**

B MASKTKASEA LKVVARCRPL SR**KEEAAGHE** **QILTMDVKLG** **QVTLR**NPR**AA** **PGELPKTFTF** **DAVYDASSKQ** **ADLYDETVRP**
LIDSVLQGFN **GTVFAYGQTG** **TGKTYTMQGT** **WVEPELRGVI** **PNAFEHIFTH** **ISRSQNQQYL** **VRASYLEIYQ** **EEIRDLLSKE**
PGKR**LELKEN** **PETGVYIKDL** **SSFVTKNVKE** **IEHVMNLGNQ** **ARAVGSTHMN** **EVSSRSHAIF** **VITVECSERG** **SDGQDHIRVG**
KLNLVDLAGS **ER**QNK**AGPNA** **AGGPATQPTA** **GGGSGSGSAS** **GSASSGERPK** **EASKINLSLS** **ALGNVIAALA** **GNRSTHIPYR**
DSKLTR**LLQD** **SLGGNAKTIM** **VATLGPASHS** **YDESLSTLR**F ANRAKNIKNK PR**VNEDPKDT** **LLREFQEEIA** **R**LK**AQLDFYF**
GKLR**NIELIC** **QENEGENDPV** **LQRIVDILYA** **TDETTSENLY** **FQGASHHHHH** **HHH**

Figure S6. *Coverage maps for KIF3C mass spectrometry analysis*. Sequences of the KIF3C with high confidence coverage in the mass spectrometry analysis denoted as black residue codes in systems with (A; 88.0% coverage) and without (B; 91.6% coverage) AMP-PNP.

A **MRECISVHVG QAGVQMGNAC WELYCLEHGI QPDGQMPSDK TIGGGDDSFT TFFCETGAGK** HVPR**AVFVDL EPTVIDEIRN**
GPYRQLFHPE QLITGKEDAA NNYARGHYTI GKEIIDPVLD RIR**KLSDQCT GLQGFLVFHS FGGGTGSGFT SLLMERLSVD**
YGKKSKLEFS IYPAPQVSTA VVEPYNSILT THTTLEHSDC AFMVDNEAIY DICR**RNLDIE RPTYTNLNRL ISQIVSSITA**
SLRFDGALNV DLTEFQTNLV PYPRIHFPLA TYAPVISAEK AYHEQLSVAE ITNACFEPAN QMVKCDPRHG K**YMACCLLYR**
GDVVPK**DVNA AIAAIK**TK**RS IQFVDWCPTG FKVGINYQPP TVVPGGDLAK** VQR**AVCMLSN TTAIAEAWAR LDHKFDLMYA**
KRAFVHWYVG EGMEEGEFSE AREDMAALEK DYEEVGIDSY EDEDEGEE

B MR**ECISVHVG QAGVQMGNAC WELYCLEHGI QPDGQMPSDK TIGGGDDSFT TFFCETGAGK** HVPR**AVFVDL EPTVIDEIRN**
GPYRQLFHPE QLITGKEDAA NNYARGHYTI GKEIIDPVLD RIRK**LSDQCT GLQGFLVFHS FGGGTGSGFT SLLMERLSVD**
YGKKSKLEFS IYPAPQVSTA VVEPYNSILT THTTLEHSDC AFMVDNEAIY DICR**RNLDIE RPTYTNLNRL ISQIVSSITA**
SLRFDGALNV DLTEFQTNLV PYPRIHFPLA TYAPVISAEK AYHEQLSVAE ITNACFEPAN QMVKCDPR**HG KYMACCLLYR**
GDVVPK**DVNA AIAAIK**TK**RS IQFVDWCPTG FKVGINYQPP TVVPGGDLAK VQRAVCMLSN TTAIAEAWAR LDHKFDLMYA**
KR**AFVHWYVG EGMEEGEFSE AREDMAALEK** DYEEVGIDSY EDEDEGEE

C MR**ECISVHVG QAGVQMGNAC WELYCLEHGI QPDGQMPSDK TIGGGDDSFT TFFCETGAGK** HVPR**AVFVDL EPTVIDEIR**N
GPYR**QLFHPE QLITGKEDAA NNYARGHYTI GKEIIDPVLD R**IRK**LSDQCT GLQGFLVFHS FGGGTGSGFT SLLMERLSVD**
YGKKSKLEFS IYPAPQVSTA VVEPYNSILT THTTLEHSDC AFMVDNEAIY DICR**RNLDIE RPTYTNLNRL ISQIVSSITA**
SLRFDGALNV DLTEFQTNLV PYPRIHFPLA TYAPVISAEK AYHEQLSVAE ITNACFEPAN QMVKCDPRHG K**YMACCLLYR**
GDVVPKDVNA AIAAIKTKRS IQFVDWCPTG FKVGINYQPP TVVPGGDLAK VQR**AVCMLSN TTAIAEAWAR LDHKFDLMYA**
KRAFVHWYVG EGMEEGEFSE AREDMAALEK DYEEVGIDSY EDEDEGEE

D MR**ECISVHVG QAGVQMGNAC WELYCLEHGI QPDGQMPSDK TIGGGDDSFT TFFCETGAGK** HVPR**AVFVDL EPTVIDEIRN**
GPYRQLFHPE QLITGKEDAA NNYARGHYTI GKEIIDPVLD RIRK**LSDQCT GLQGFLVFHS FGGGTGSGFT SLLMERLSVD**
YGKKSKLEFS IYPAPQVSTA VVEPYNSILT THTTLEHSDC AFMVDNEAIY DICR**RNLDIE RPTYTNLNRL ISQIVSSITA**
SLRFDGALNV DLTEFQTNLV PYPRIHFPLA TYAPVISAEK AYHEQLSVAE ITNACFEPAN QMVKCDPRHG K**YMACCLLYR**
GDVVPKDVNA AIAAIKTKRS IQFVDWCPTG FKVGINYQPP TVVPGGDLAK VQR**AVCMLSN TTAIAEAWAR LDHKFDLMYA**
KRAFVHWYVG EGMEEGEFSE AREDMAALEK DYEEVGIDSY EDEDEGEE

E MR**ECISVHVG QAGVQMGNAC WELYCLEHGI QPDGQMPSDK TIGGGDDSFT TFFCETGAGK** HVPR**AVFVDL EPTVIDEIRN**
GPYRQLFHPE QLITGKEDAA NNYARGHYTI GKEIIDPVLD RIR**KLSDQCT GLQGFLVFHS FGGGTGSGFT SLLMERLSVD**
YGKKSKLEFS IYPAPQVSTA VVEPYNSILT THTTLEHSDC AFMVDNEAIY DICR**RNLDIE RPTYTNLNRL ISQIVSSITA**
SLRFDGALNV DLTEFQTNLV PYPRIHFPLA TYAPVISAEK AYHEQLSVAE ITNACFEPAN QMVKCDPRHG K**YMACCLLYR**
GDVVPK**DVNA AIAAIKTKRS IQFVDWCPTG FKVGINYQPP TVVPGGDLAK** VQR**AVCMLSN TTAIAEAWAR LDHKFDLMYA**
KRAFVHWYVG EGMEEGEFSE AREDMAALEK DYEEVGIDSY EDEDEGEE

F MR**ECISVHVG QAGVQMGNAC WELYCLEHGI QPDGQMPSDK TIGGGDDSFT TFFCETGAGK** HVPR**AVFVDL EPTVIDEIR**N
GPYR**QLFHPE QLITGKEDAA NNYARGHYTI GKEIIDPVLD R**IRK**LSDQCT GLQGFLVFHS FGGGTGSGFT SLLMERLSVD**
YGKKSKLEFS IYPAPQVSTA VVEPYNSILT THTTLEHSDC AFMVDNEAIY DICR**RNLDIE RPTYTNLNRL ISQIVSSITA**
SLRFDGALNV DLTEFQTNLV PYPRIHFPLA TYAPVISAEK AYHEQLSVAE ITNACFEPAN QMVKCDPRHG **KYMACCLLYR**
GDVVPKDVNA AIAAIKTKRS IQFVDWCPTG FKVGINYQPP TVVPGGDLAK VQR**AVCMLSN TTAIAEAWAR LDHKFDLMYA**
KRAFVHWYVG EGMEEGEFSE AREDMAALEK DYEEVGIDSY EDEDEGEE

Figure S7. *Coverage maps for Tubulin alpha-4A mass spectrometry analysis.* Sequences of the Tubulin alpha-4A in K439 (A-B), KIF3AB (C-D), and KIF3AC (E-F) systems with high confidence coverage in the mass spectrometry analysis denoted as black residue codes in systems with (A; 79.5% coverage, C; 79.5% coverage, E; 79.5% coverage) and without (B; 80.0% coverage, D; 80.6% coverage, F; 79.7% coverage) AMPPNP.

A **MREIVHLQAG QCGNQIGAKF WEVISDEHGI DPTGTYHGDS DLQLERINVY YNEATGGNYV PRAVLVDLEP GTMDSVRSGP**
FGQIFRPDNF VFGQSGAGNN WAKGHYTEGA ELVDAVLDVV RKEAESCDCL QGFQLTHSLG GGTGSGMGTL LISKIREEFP
DRIMNTFSVV PSPKVSDTVV EPYNATLSVH QLVENTDETY CIDNEALYDI CFR**TLKLTTP TYGDLNHLVS ATMSGVTTCL**
RFPGQLNADL RKLAVNMVPF PRLHFFMPGF APLTSRGSQQ YR**ALTVPELT QQMFDAKNMM AACDPRHGRY LTVAAVFR**GR
MSMK**EVDEQM LSVQSKNSSY FVEWIPNNVK TAVCDIPPRG LKMAATFIGN STAIQELFKR ISEQFTAMFR R**KAFLHWYTG
EGMDEMEFTE AESNMNDLVS EYQQYQDATA EEGEFEEEAE EEVA

B **MREIVHLQAG QCGNQIGAKF WEVISDEHGI DPTGTYHGDS DLQLERINVY YNEATGGKYV PRAVLVDLEP GTMDSVRSGP**
FGQIFRPDNF VFGQSGAGNN WAKGHYTEGA ELVDSVLDVV RKEAESCDCL QGFQLTHSLG GGTGSGMGTL LISKIREEYP
DRIMNTFSVV PSPKVSDTVV EPYNATLSVH QLVENTDETY CIDNEALYDI CFR**TLKLTTP TYGDLNHLVS ATMSGVTTCL**
RFPGQLNADL RKLAVNMVPF PRLHFFMPGF APLTSRGSQQ YR**ALTVPELT QQMFDAKNMM AACDPR**HGR**Y LTVAAVFRGR**
MSMKEVDEQM LNVQNKNSSY FVEWIPNNVK TAVCDIPPRG LKMSATFIGN STAIQELFKR ISEQFTAMFR RKAFLHWYTG
EGMDEMEFTE AESNMNDLVS EYQQYQDATA EEEGEFEEEA EEEVA

C **MREIVHLQAG QCGNQIGAKF WEVISDEHGI DPTGTYHGDS DLQLERINVY YNEATGGK**YV PR**AVLVDLEP GTMDSVRSGP**
FGQIFRPDNF VFGQSGAGNN WAKGHYTEGA ELVDSVLDVV RKEAESCDCL QGFQLTHSLG GGTGSGMGTL LISKIREEYP
DRIMNTFSVV PSPKVSDTVV EPYNATLSVH QLVENTDETY CIDNEALYDI CFR**TLKLTTP TYGDLNHLVS ATMSGVTTCL**
RFPGQLNADL RKLAVNMVPF PRLHFFMPGF APLTSRGSQQ YR**ALTVPELT QQMFDAKNMM AACDPR**HGR**Y LTVAAVFRGR**
MSMKEVDEQM LNVQNKNSSY FVEWIPNNVK TAVCDIPPRG LKMSATFIGN STAIQELFKR ISEQFTAMFR RKAFLHWYTG
EGMDEMEFTE AESNMNDLVS EYQQYQDATA EEEGEFEEEA EEEVA

D **MREIVHLQAG QCGNQIGAKF WEVISDEHGI DPTGTYHGDS DLQLERINVY YNEATGGKYV PRAVLVDLEP GTMDSVRSGP**
FGQIFRPDNF VFGQSGAGNN WAKGHYTEGA ELVDSVLDVV RKEAESCDCL QGFQLTHSLG GGTGSGMGTL LISKIREEYP
DRIMNTFSVV PSPKVSDTVV EPYNATLSVH QLVENTDETY CIDNEALYDI CFR**TLKLTTP TYGDLNHLVS ATMSGVTTCL**
RFPGQLNADL RKLAVNMVPF PRLHFFMPGF APLTSRGSQQ YR**ALTVPELT QQMFDAKNMM AACDPR**HGR**Y LTVAAVFRGR**
MSMKEVDEQM LNVQNKNSSY FVEWIPNNVK TAVCDIPPRG LKMSATFIGN STAIQELFKR ISEQFTAMFR RKAFLHWYTG
EGMDEMEFTE AESNMNDLVS EYQQYQDATA EEEGEFEEEA EEEVA

E **MREIVHLQAG QCGNQIGAKF WEVISDEHGI DPTGTYHGDS DLQLERINVY YNEATGGKYV PRAVLVDLEP GTMDSVRSGP**
FGQIFRPDNF VFGQSGAGNN WAKGHYTEGA ELVDSVLDVV RKEAESCDCL QGFQLTHSLG GGTGSGMGTL LISKIREEYP
DRIMNTFSVV PSPKVSDTVV EPYNATLSVH QLVENTDETY CIDNEALYDI CFR**TLKLTTP TYGDLNHLVS ATMSGVTTCL**
RFPGQLNADL RKLAVNMVPF PRLHFFMPGF APLTSRGSQQ YR**ALTVPELT QQMFDAKNMM AACDPR**HGR**Y LTVAAVFRGR**
MSMKEVDEQM LNVQNKNSSY FVEWIPNNVK TAVCDIPPRG LKMSATFIGN STAIQELFKR ISEQFTAMFR RKAFLHWYTG
EGMDEMEFTE AESNMNDLVS EYQQYQDATA EEEGEFEEEA EEEVA

F **MREIVHLQAG QCGNQIGAKF WEVISDEHGI DPTGTYHGDS DLQLERINVY YNEATGGK**YV PR**AVLVDLEP GTMDSVRSGP**
FGQIFRPDNF VFGQSGAGNN WAKGHYTEGA ELVDSVLDVV RKEAESCDCL QGFQLTHSLG GGTGSGMGTL LISKIREEYP
DRIMNTFSVV PSPKVSDTVV EPYNATLSVH QLVENTDETY CIDNEALYDI CFRTLK**LTTP TYGDLNHLVS ATMSGVTTCL**
RFPGQLNADL RKLAVNMVPF PRLHFFMPGF APLTSRGSQQ YR**ALTVPELT QQMFDAKNMM AACDPR**HGR**Y LTVAAVFRGR**
MSMKEVDEQM LNVQNKNSSY FVEWIPNNVK TAVCDIPPRG LKMSATFIGN STAIQELFKR ISEQFTAMFR RKAFLHWYTG
EGMDEMEFTE AESNMNDLVS EYQQYQDATA EEEGEFEEEA EEEVA

Figure S8. *Coverage maps for Tubulin beta-4B mass spectrometry analysis.* Sequences of the Tubulin beta-4B in K439 (A-B), KIF3AB (C-D), and KIF3AC (E-F) systems with high confidence coverage in the mass spectrometry analysis denoted as black residue codes in systems with (A; 76.6% coverage, C; 76.4% coverage, E; 77.0% coverage) and without (B; 77.5% coverage, D; 77.3% coverage, F; 76.3% coverage) AMP-PNP.

A

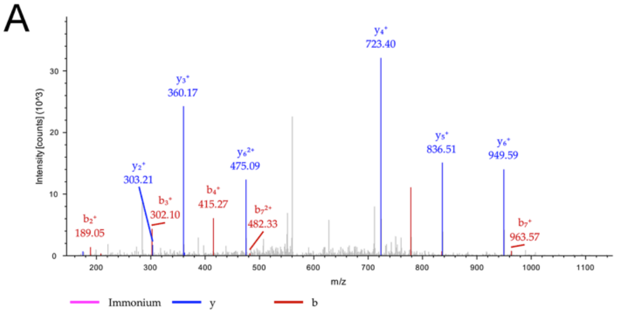

#1	Immonium	b+	b2+	Seq.	y+	y2+	#2
1	60.04439	88.03931	44.52329	S			8
2	74.06004	189.08699	95.04713	T	1050.55945	525.78336	7
3	86.09643	302.17106	151.58917	L	949.51177	475.25952	6
4	86.09643	415.25513	208.13120	L	836.42770	418.71749	5
5	120.08078	778.39975	389.70351	F-AziPm	723.34363	362.17545	4
6	30.03383	835.42122	418.21425	G	360.19901	180.60314	3
7	101.07094	963.47980	482.24354	Q	303.17754	152.09241	2
8				R	175.11896	88.06312	1

B

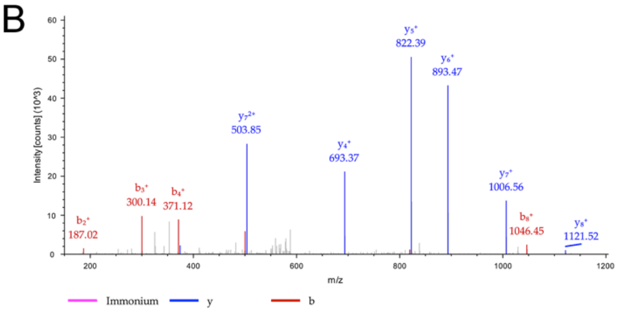

#1	Immonium	b+	b2+	Seq.	y+	y2+	#2
1	44.04948	72.04440	36.52584	A			9
2	88.03931	187.07135	94.03931	D	1121.51594	561.26161	8
3	86.09643	300.15542	150.58135	L	1006.48899	503.74813	7
4	44.04948	371.19254	186.09991	A	893.40492	447.20610	6
5	102.05496	500.23514	250.62121	E	822.36780	411.68754	5
6	76.02155	819.32053	410.16390	C-AziPm	693.32520	347.16624	4
7	87.05529	933.36346	467.18537	N	374.23981	187.62354	3
8	86.09643	1046.44753	523.72740	I	260.19688	130.60208	2
9				K	147.11281	74.06004	1

C

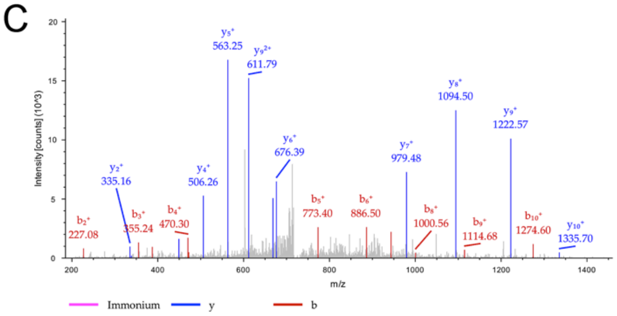

#1	Immonium	b+	b2+	Seq.	y+	y2+	#2
1	86.09643	114.09135	57.54931	I			11
2	86.09643	227.17542	114.09135	L	1335.59739	668.30233	10
3	101.07094	355.23400	178.12064	Q	1222.51332	611.76030	9
4	88.03931	470.26095	235.63411	D	1094.45474	547.73101	8
5	60.04439	773.36918	387.18823	S-AziPm	979.42779	490.21753	7
6	86.09643	886.45325	443.73026	L	676.31956	338.66342	6
7	30.03383	943.47472	472.24100	G	563.23549	282.12138	5
8	30.03383	1000.49619	500.75173	G	506.21402	253.61065	4
9	87.05529	1114.53912	557.77320	N	449.19255	225.09991	3
10	76.02155	1274.56977	637.78852	C-Carbamidomethyl	335.14962	168.07845	2
11				R	175.11896	88.06312	1

D

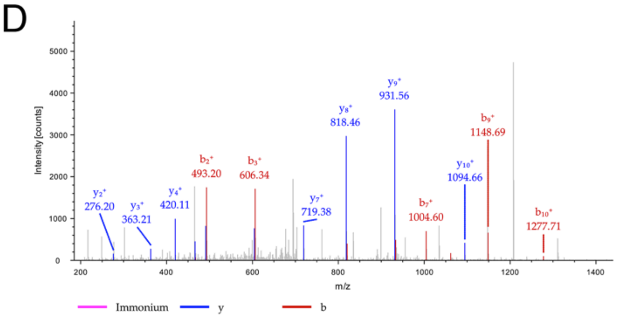

#1	Immonium	b+	b2+	Seq.	y+	y2+	#2
1	86.09643	330.16755	165.58741	L-AziPm			11
2	136.07568	493.23087	247.11907	Y	1094.57286	547.79007	10
3	86.09643	606.31494	303.66111	L	931.50954	466.25841	9
4	72.08078	705.38336	353.19532	V	818.42547	409.71637	8
5	88.03931	820.41031	410.70879	D	719.35705	360.18216	7
6	86.09643	933.49438	467.25083	L	604.33010	302.66869	6
7	44.04948	1004.53150	502.76939	A	491.24603	246.12665	5
8	30.03383	1061.55297	531.28012	G	420.20891	210.60809	4
9	60.04439	1148.58500	574.79614	S	363.18744	182.09736	3
10	102.05496	1277.62760	639.31744	E	276.15541	138.58134	2
11				K	147.11281	74.06004	1

Figure S9. *Identification of meta-azipropofol (AziPm) adducts within K439 in samples containing microtubules and without (A) or with AMP-PNP (B-D)*. (A) (top) Mass spectrum of the ^{314}STLL**F**GQR^{321} peptide, which contains an AziP*m* adduct at F318 and (bottom) fragment table showing the fragmentation of the ^{314}STLL**F**GQR^{321} photolabeled peptide. (B) (top) Mass spectrum of the ^{2}ADLAE**C**NIK^{10} peptide, which contains an AziP*m* adduct at C7 and (bottom) fragment table showing the fragmentation of the ^{2}ADLAE**C**NIK^{10} photolabeled peptide. (C) (top) Mass spectrum of the ^{285}ILQD**S**LGGNC^{295} peptide, which contains an AziP*m* adduct at S289 and (bottom) fragment table showing the fragmentation of the ^{285}ILQD**S**LGGNC^{295} photolabeled peptide. (D) (top) Mass spectrum of the ^{227}**L**YLVDLAGSEK^{237} peptide, which contains an AziP*m* adduct at L227 and (bottom) fragment table showing the fragmentation of the ^{227}**L**YLVDLAGSEK^{237} photolabeled peptide. Detected identified immonium (magenta), a, b (red) and y (blue) ions are labeled accordingly. Residues detected with a modification are noted.

A

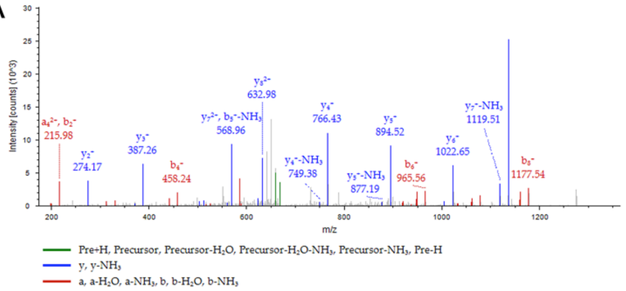

C

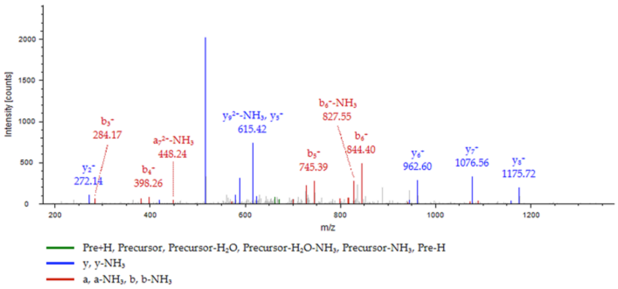

B

#1	a+	a2+	b+	b2+	Seq.	y+	y2+	#2
1	60.04439	30.52583	88.03931	44.52329	S			9
2	188.10297	94.55512	216.09789	108.55258	Q	1264.62964	632.81846	8
3	302.14590	151.57659	330.14082	165.57405	N	1136.57106	568.78917	7
4	430.20448	215.60588	458.19940	229.60334	Q	1022.52813	511.76770	6
5	558.26306	279.63517	586.25798	293.63263	Q	894.46955	447.73841	5
6	937.40258	469.20493	965.39750	483.20239	Y-AziPm	766.41097	383.70912	4
7	1050.48665	525.74696	1078.48157	539.74442	L	387.27145	194.13936	3
8	1149.55507	575.28117	1177.54999	589.27863	V	274.18738	137.59733	2
9					R	175.11896	88.06312	1

D

#1	a+	a2+	b+	b2+	Seq.	y+	y2+	#2
1	86.09643	43.55185	114.09135	57.54931	L			10
2	157.13355	79.07041	185.12847	93.06787	A	1246.62651	623.81689	9
3	256.20197	128.60462	284.19689	142.60208	V	1175.58939	588.29833	8
4	370.24490	185.62609	398.23982	199.62355	N	1076.52097	538.76412	7
5	717.36160	359.18444	745.35652	373.18190	M-AziPm	962.47804	481.74266	6
6	816.43002	408.71865	844.42494	422.71611	V	615.36134	308.18431	5
7	913.48279	457.24503	941.47771	471.24249	P	516.29292	258.65010	4
8	1060.55121	530.77924	1088.54613	544.77670	F	419.24015	210.12371	3
9	1157.60398	579.30563	1185.59890	593.30309	P	272.17173	136.58950	2
10					R	175.11896	88.06312	1

Figure S10. *Identification of meta-azipropofol (AziPm) adducts within KIF3B (A-B) or Tubulin beta-4B (C-D) in samples containing microtubules and AMP-PNP.* (A) Mass spectrum of the ^{133}SQNQQ**Y**LVR^{141} peptide, which contains an AziP*m* adduct at Y138 and (B) fragment table showing the fragmentation of the ^{133}SQNQQ**Y**LVR^{141} photolabeled peptide. (C) Mass spectrum of the ^{253}LAVN**M**VPFPR^{262} peptide, which contains an AziP*m* adduct at M257 and (D) fragment table showing the fragmentation of the ^{253}LAVN**M**VPFPR^{262} photolabeled peptide. Detected identified precursor (green), a, b (red) and y (blue) ions are labeled accordingly. Residues detected with a modification are noted.

A

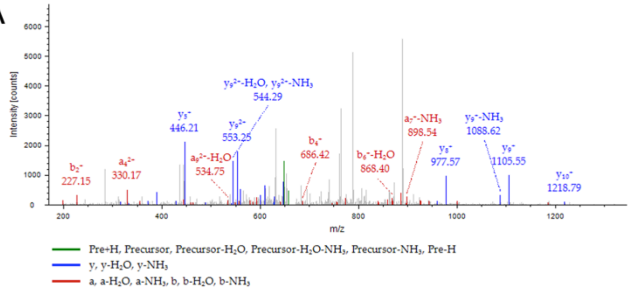

C

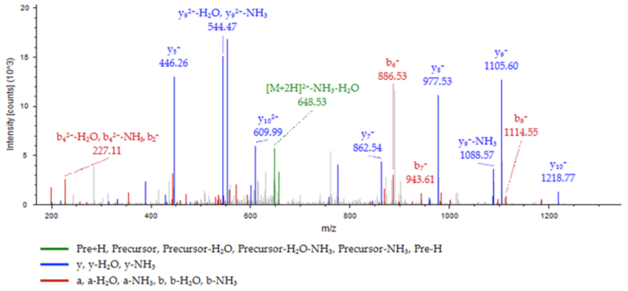

B

#1	a⁺	a²⁺	b⁺	b²⁺	Seq.	y⁺	y²⁺	#2
1	86.09643	43.55185	114.09135	57.54931	L			11
2	199.18050	100.09389	227.17542	114.09135	L	1218.59770	609.80249	10
3	327.23908	164.12318	355.23400	178.12064	Q	1105.51363	553.26045	9
4	658.34223	329.67475	686.33715	343.67221	D-AziPm	977.45505	489.23116	8
5	745.37426	373.19077	773.36918	387.18823	S	646.35190	323.67959	7
6	858.45833	429.73280	886.45325	443.73026	L	559.31987	280.16357	6
7	915.47980	458.24354	943.47472	472.24100	G	446.23580	223.62154	5
8	972.50127	486.75427	1000.49619	500.75173	G	389.21433	195.11080	4
9	1086.54420	543.77574	1114.53912	557.77320	N	332.19286	166.60007	3
10	1157.58132	579.29430	1185.57624	593.29176	A	218.14993	109.57860	2
11					K	147.11281	74.06004	1

D

#1	a⁺	a²⁺	b⁺	b²⁺	Seq.	y⁺	y²⁺	#2
1	86.09643	43.55185	114.09135	57.54931	L			11
2	199.18050	100.09389	227.17542	114.09135	L	1218.59770	609.80249	10
3	327.23908	164.12318	355.23400	178.12064	Q	1105.51363	553.26045	9
4	442.26603	221.63665	470.26095	235.63411	D	977.45505	489.23116	8
5	529.29806	265.15267	557.29298	279.15013	S	862.42810	431.71769	7
6	858.45833	429.73280	886.45325	443.73026	L-AziPm	775.39607	388.20167	6
7	915.47980	458.24354	943.47472	472.24100	G	446.23580	223.62154	5
8	972.50127	486.75427	1000.49619	500.75173	G	389.21433	195.11080	4
9	1086.54420	543.77574	1114.53912	557.77320	N	332.19286	166.60007	3
10	1157.58132	579.29430	1185.57624	593.29176	A	218.14993	109.57860	2
11					K	147.11281	74.06004	1

E

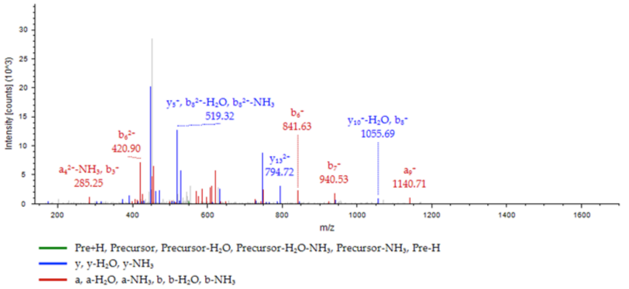

F

#1	a⁺	a²⁺	a³⁺	b⁺	b²⁺	b³⁺	Seq.	y⁺	y²⁺	y³⁺	#2
1	72.08078	36.54403	24.69845	100.07570	50.54149	34.03008	V				14
2	129.10225	65.05476	43.70560	157.09717	79.05222	53.03724	G	1587.83533	794.42130	529.94996	13
3	257.19722	129.10225	86.40393	285.19214	143.09971	95.73556	K	1530.81386	765.91057	510.94280	12
4	586.35749	293.68238	196.12402	614.35241	307.67984	205.45565	L-AziPm	1402.71889	701.86308	468.24448	11
5	700.40042	350.70385	234.13833	728.39534	364.70131	243.46996	N	1073.55862	537.28295	358.52439	10
6	813.48449	407.24588	271.83302	841.47941	421.24334	281.16465	L	959.51569	480.26148	320.51008	9
7	912.55291	456.78009	304.85582	940.54783	470.77755	314.18746	V	846.43162	423.71945	282.81539	8
8	1027.57986	514.29357	343.19814	1055.57478	528.29103	352.52978	D	747.36320	374.18524	249.79258	7
9	1140.66393	570.83560	380.89283	1168.65885	584.83306	390.22447	L	632.33625	316.67176	211.45027	6
10	1211.70105	606.35416	404.57187	1239.69597	620.35162	413.90351	A	519.25218	260.12973	173.75558	5
11	1268.72252	634.86490	423.57903	1296.71744	648.86236	432.91066	G	448.21506	224.61117	150.07654	4
12	1355.75455	678.38091	452.58970	1383.74947	692.37837	461.92134	S	391.19359	196.10043	131.06938	3
13	1484.79715	742.90221	495.60390	1512.79207	756.89967	504.93554	E	304.16156	152.58442	102.05870	2
14							R	175.11896	88.06312	59.04450	1

Figure S11. *Identification of meta-azipropofol (AziPm) adducts within KIF3C in samples containing microtubules and without AMP-PNP*. (A) Mass spectrum of the ^{327}LLQ**D**SLGGNAK^{337} peptide, which contains an AziP*m* adduct at D330 and (B) fragment table showing the fragmentation of the ^{327}LLQ**D**SLGGNAK^{337} photolabeled peptide. (C) Mass spectrum of the ^{327}LLQDS**L**GGNAK^{337} peptide, which contains an AziP*m* adduct at L332 and (D) fragment table showing the fragmentation of the ^{327}LLQDS**L**GGNAK^{337} photolabeled peptide. (E) Mass spectrum of the ^{239}VGK**L**NLVDLAGSER^{252} peptide, which contains an AziP*m* adduct at L242 and (F) fragment table showing the fragmentation of the ^{239}VGK**L**NLVDLAGSER^{252} photolabeled peptide. Detected identified precursor ion (green), a, b (red) and y (blue) ions are labeled accordingly. Residues detected with a modification are noted.

A

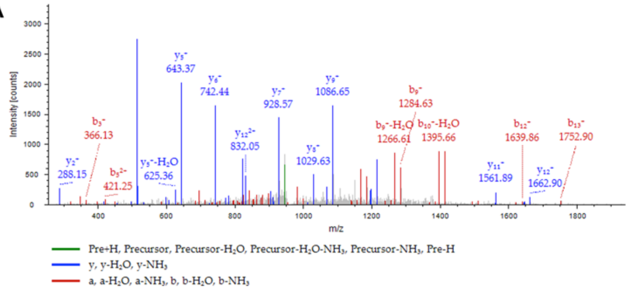

B

#1	a⁺	a²⁺	b⁺	b²⁺	Seq.	y⁺	y²⁺	#2
1	74.06004	37.53366	102.05496	51.53112	T			14
2	237.12336	119.06532	265.11828	133.06278	Y	1825.84417	913.42572	13
3	338.17104	169.58916	366.16596	183.58662	T	1662.78085	831.89406	12
4	685.28774	343.14751	713.28266	357.14497	M-AziPm	1561.73317	781.37022	11
5	813.34632	407.17680	841.34124	421.17426	Q	1214.61647	607.81187	10
6	870.36779	435.68753	898.36271	449.68499	G	1086.55789	543.78258	9
7	971.41547	486.21137	999.41039	500.20883	T	1029.53642	515.27185	8
8	1157.49479	579.25103	1185.48971	593.24849	W	928.48874	464.74801	7
9	1256.56321	628.78524	1284.55813	642.78270	V	742.40942	371.70835	6
10	1385.60581	693.30654	1413.60073	707.30400	E	643.34100	322.17414	5
11	1482.65858	741.83293	1510.65350	755.83039	P	514.29840	257.65284	4
12	1611.70118	806.35423	1639.69610	820.35169	E	417.24563	209.12645	3
13	1724.78525	862.89626	1752.78017	876.89372	L	288.20303	144.60515	2
14					R	175.11896	88.06312	1

C

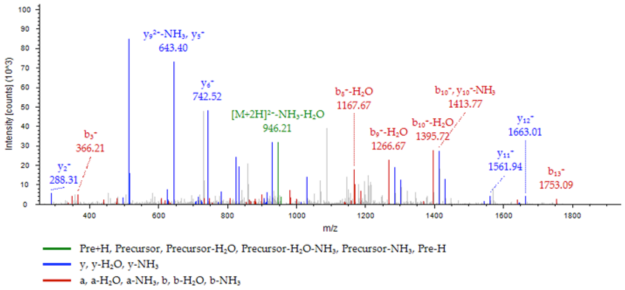

D

#1	a⁺	a²⁺	b⁺	b²⁺	Seq.	y⁺	y²⁺	#2
1	74.06004	37.53366	102.05496	51.53112	T			14
2	237.12336	119.06532	265.11828	133.06278	Y	1825.84417	913.42572	13
3	338.17104	169.58916	366.16596	183.58662	T	1662.78085	831.89406	12
4	469.21154	235.10941	497.20646	249.10687	M	1561.73317	781.37022	11
5	597.27012	299.13870	625.26504	313.13616	Q	1430.69267	715.84997	10
6	870.36779	435.68753	898.36271	449.68499	G-AziPm	1302.63409	651.82068	9
7	971.41547	486.21137	999.41039	500.20883	T	1029.53642	515.27185	8
8	1157.49479	579.25103	1185.48971	593.24849	W	928.48874	464.74801	7
9	1256.56321	628.78524	1284.55813	642.78270	V	742.40942	371.70835	6
10	1385.60581	693.30654	1413.60073	707.30400	E	643.34100	322.17414	5
11	1482.65858	741.83293	1510.65350	755.83039	P	514.29840	257.65284	4
12	1611.70118	806.35423	1639.69610	820.35169	E	417.24563	209.12645	3
13	1724.78525	862.89626	1752.78017	876.89372	L	288.20303	144.60515	2
14					R	175.11896	88.06312	1

E

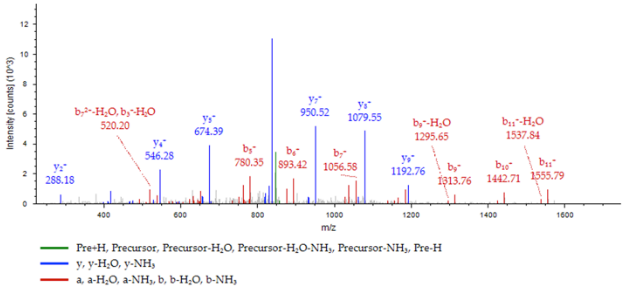

F

#1	a⁺	a²⁺	b⁺	b²⁺	Seq.	y⁺	y²⁺	#2
1	44.04948	22.52838	72.04440	36.52584	A			12
2	347.15771	174.08249	375.15263	188.07995	S-AziPm	1658.79242	829.89985	11
3	510.22103	255.61415	538.21595	269.61161	Y	1355.68419	678.34573	10
4	623.30510	312.15619	651.30002	326.15365	L	1192.62087	596.81407	9
5	752.34770	376.67749	780.34262	390.67495	E	1079.53680	540.27204	8
6	865.43177	433.21952	893.42669	447.21698	I	950.49420	475.75074	7
7	1028.49509	514.75118	1056.49001	528.74864	Y	837.41013	419.20870	6
8	1156.55367	578.78047	1184.54859	592.77793	Q	674.34681	337.67704	5
9	1285.59627	643.30177	1313.59119	657.29923	E	546.28823	273.64775	4
10	1414.63887	707.82307	1442.63379	721.82053	E	417.24563	209.12645	3
11	1527.72294	764.36511	1555.71786	778.36257	I	288.20303	144.60515	2
12					R	175.11896	88.06312	1

G

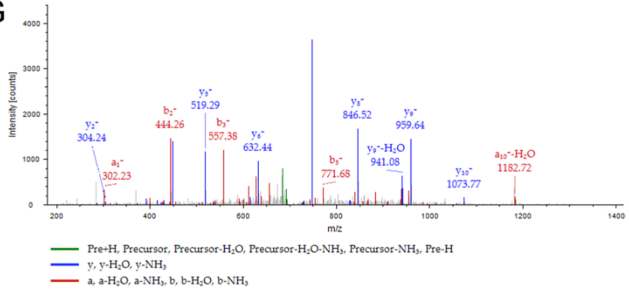

H

#1	a⁺	a²⁺	b⁺	b²⁺	Seq.	y⁺	y²⁺	#2
1	302.17263	151.58995	330.16755	165.58741	L-AziPm			11
2	416.21556	208.61142	444.21048	222.60888	N	1073.55862	537.28295	10
3	529.29963	265.15345	557.29455	279.15091	L	959.51569	480.26148	9
4	628.36805	314.68766	656.36297	328.68512	V	846.43162	423.71945	8
5	743.39500	372.20114	771.38992	386.19860	D	747.36320	374.18524	7
6	856.47907	428.74317	884.47399	442.74063	L	632.33625	316.67176	6
7	927.51619	464.26173	955.51111	478.25919	A	519.25218	260.12973	5
8	984.53766	492.77247	1012.53258	506.76993	G	448.21506	224.61117	4
9	1071.56969	536.28848	1099.56461	550.28594	S	391.19359	196.10043	3
10	1200.61229	600.80978	1228.60721	614.80724	E	304.16156	152.58442	2
11					R	175.11896	88.06312	1

I

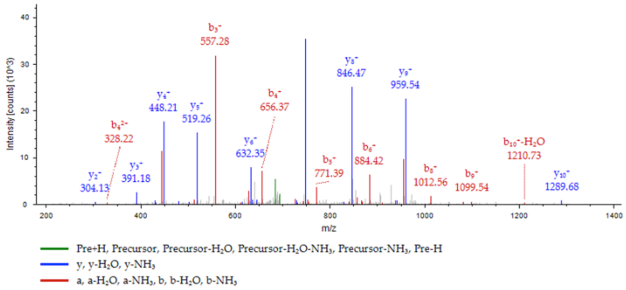

J

#1	a⁺	a²⁺	b⁺	b²⁺	Seq.	y⁺	y²⁺	#2
1	86.09643	43.55185	114.09135	57.54931	L			11
2	416.21556	208.61142	444.21048	222.60888	N-AziPm	1289.63482	645.32105	10
3	529.29963	265.15345	557.29455	279.15091	L	959.51569	480.26148	9
4	628.36805	314.68766	656.36297	328.68512	V	846.43162	423.71945	8
5	743.39500	372.20114	771.38992	386.19860	D	747.36320	374.18524	7
6	856.47907	428.74317	884.47399	442.74063	L	632.33625	316.67176	6
7	927.51619	464.26173	955.51111	478.25919	A	519.25218	260.12973	5
8	984.53766	492.77247	1012.53258	506.76993	G	448.21506	224.61117	4
9	1071.56969	536.28848	1099.56461	550.28594	S	391.19359	196.10043	3
10	1200.61229	600.80978	1228.60721	614.80724	E	304.16156	152.58442	2
11					R	175.11896	88.06312	1

Figure S12. *Identification of meta-azipropofol (AziPm) adducts within KIF3C in samples containing*

microtubules and with AMP-PNP. (A) Mass spectrum of the ^{104}TYT**M**QGTWVEPELR^{117} peptide, which contains an AziP*m* adduct at M107 and (B) fragment table showing the fragmentation of the ^{104}TYT**M**QGTWVEPELR^{117} photolabeled peptide. (C) Mass spectrum of the ^{104}TYTMQ**G**TWVEPELR^{117} peptide, which contains an AziP*m* adduct at G109 and (D) fragment table showing the fragmentation of the ^{104}TYTMQ**G**TWVEPELR^{117} photolabeled peptide. (E) Mass spectrum of the ^{143}A**S**YLEIYQEEIR^{154} peptide, which contains an AziP*m* adduct at S144 and (F) fragment table showing the fragmentation of the ^{143}A**S**YLEIYQEEIR^{154} photolabeled peptide. (G) Mass spectrum of the ^{242}**L**NLVDLAGSER^{252} peptide, which contains an AziP*m* adduct at L242 and (H) fragment table showing the fragmentation of the ^{242}**L**NLVDLAGSER^{252} photolabeled peptide. (I) Mass spectrum of the ^{242}L**N**LVDLAGSER^{252} peptide, which contains an AziP*m* adduct at N243 and (J) fragment table showing the fragmentation of the ^{242}L**N**LVDLAGSER^{252} photolabeled peptide. Detected identified precursor ion (green), a, b (red) and y (blue) ions are labeled accordingly. Residues detected with a modification are noted.

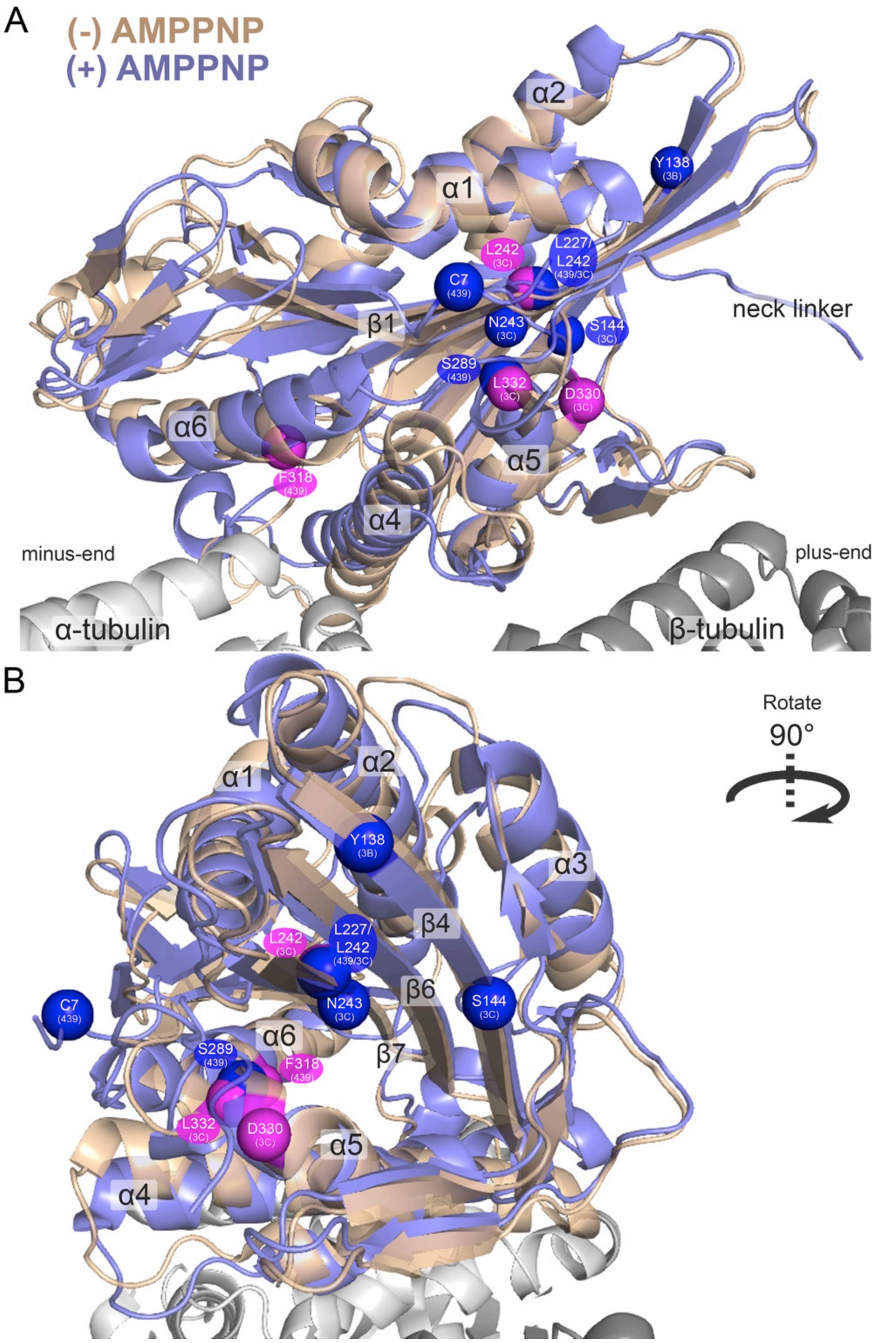

Figure S13. *Residues photolabeled by AziPm collected at the kinesin-1 (K439) and kinesin-2 (KIF3B and KIF3C) motor head subdomain interfaces in microtubule•kinesin complexes with and without AMPPNP.* (A) Side and (B) microtubule plus-end view of the x-ray crystal structure of kinesin motor head in complex with tubulin in the absence of nucleotide (PDB ID: 4LNU; wheat) (2) and ADP-AlF_4^- -bound kinesin motor head in complex with tubulin (PDB ID: 4HNA; blue) (3) respresenting photolabeled microtubule•kinesin complexes without (-) and with (+) AMPPNP respectively. Kinesin motor domain sequences were aligned with K439, KIF3B, and KIF3C. The alpha-carbon of photoaffinity labeled residues are shown as spheres and labeled with associated kinesin, K439 (439), KIF3B (3B), and KIF3C (3C). Photoaffinity labeled residues identified in microtubule•kinesin complexes without AMPPNP within K439 and KIF3C are colored magenta. The common allosteric site identified for K439, KIF3B and KIF3C in the AMPPNP-bound microtubule•kinesin complexes state are colored in blue.

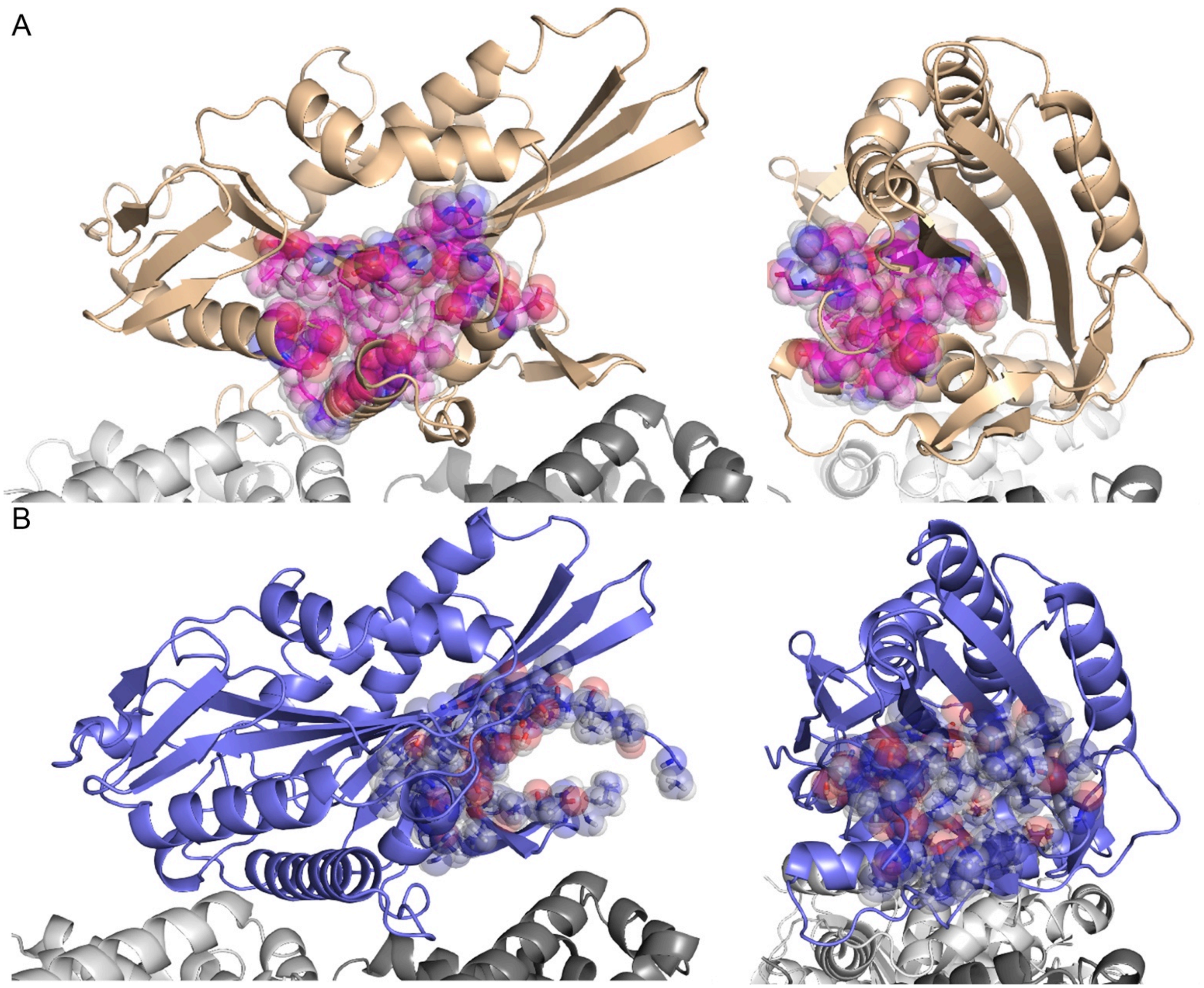

Figure S14. *Side (left) and plus-end (right) views CASTp* (4) *predicted pockets within the X-ray crystal structures of kinesin motor domain without nucleotide (PDB ID: 4LNU)* (2) *(A) or with ADP-AlF$_4^-$ (PDB ID: 4HNA)* (3) *(B) in complex with tubulin with residues mutated to match the K439 sequence.* Residues detected to contribute to binding cavity are shown in spherical and stick representations and were used to define the flexible residues and docking site for AutoDockVina (5) experiments. The pocket volume within the kinesin tubulin complex without nucleotide (A) was predicted as 307 $Å^3$ (Connolly's surface). The pocket volume within the kinesin tubulin complex with ADP-AlF$_4^-$ (B) was predicted as 1690 $Å^3$ (Connolly's surface).

REFERENCES

1. Bensel, B. M., Guzik-Lendrum, S., Masucci, E. M., Woll, K. A., Eckenhoff, R. G., and Gilbert, S. P. (2017) Common general anesthetic propofol impairs kinesin processivity. *Proc. Natl. Acad. Sci. U. S. A.* **144**, E4281–E4287
2. Cao, L., Wang, W., Jiang, Q., Wang, C., Knossow, M., and Gigant, B. (2014) The structure of apo-kinesin bound to tubulin links the nucleotide cycle to movement. *Nat. Commun.* **5**, 5364
3. Gigant, B., Wang, W., Dreier, B., Jiang, Q., Pecqueur, L., Pluckthun, A., Wang, C., and Knossow, M. (2013) Structure of a kinesin-tubulin complex and implications for kinesin motility. *Nat. Struct. Mol. Biol.* **20**, 1001–1007
4. Dundas, J., Ouyang, Z., Tseng, J., Binkowski, A., Turpaz, Y., and Liang, J. (2006) CASTp: computed atlas of surface topography of proteins with structural and topographical mapping of functionally annotated residues. *Nucleic Acids Res.* **34**, W116-8
5. Trott, O., and Olson, A. J. (2010) AutoDock Vina: improving the speed and accuracy of docking with a new scoring function, efficient optimization, and multithreading. *J. Comput. Chem.* **31**, 455–61
